# Coordination of signalling networks and tumorigenic properties by ABL in glioblastoma cells

**DOI:** 10.18632/oncotarget.12546

**Published:** 2016-10-09

**Authors:** Fabienne Lamballe, Sara Toscano, Filippo Conti, Maria Arechederra, Nathalie Baeza, Dominique Figarella-Branger, Françoise Helmbacher, Flavio Maina

**Affiliations:** ^1^ Aix-Marseille Université, CNRS, Developmental Biology Institute of Marseille (IBDM), Parc Scientifique de Luminy, Marseille, France; ^2^ Aix-Marseille Université, Inserm, CRO2 UMR S911, Marseille, France

**Keywords:** ABL, migration and invasion, tumorigenicity and self-renewal, RTK signalling, glioblastomas

## Abstract

The cytoplasmic tyrosine kinase ABL exerts positive or negative effects in solid tumours according to the cellular context, thus functioning as a “switch modulator”. The therapeutic effects of drugs targeting a set of signals encompassing ABL have been explored in several solid tumours. However, the net contribution of ABL inhibition by these agents remains elusive as these drugs also act on other signalling components. Here, using glioblastoma (GBM) as a cellular paradigm, we report that ABL inhibition exacerbates mesenchymal features as highlighted by down-regulation of epithelial markers and up-regulation of mesenchymal markers. Cells with permanent ABL inhibition exhibit enhanced motility and invasive capabilities, while proliferation and tumorigenic properties are reduced. Intriguingly, permanent ABL inhibition also interferes with GBM neurosphere formation and with expression of stemness markers in sphere-cultured GBM cells. Furthermore, we show that the molecular and biological characteristics of GBM cells with impaired ABL are reversible by restoring ABL levels, thus uncovering a remarkable plasticity of GBM cells to ABL threshold. A phospho-signalling screen revealed that loss of tumorigenic and self-renewal properties in GBM cells under permanent ABL inhibition coincide with drastic changes in the expression and/or phosphorylation levels of multiple signalling components. Our findings identify ABL as a crucial player for migration, invasion, proliferation, tumorigenic, and stem-cell like properties of GBM cells. Taken together, this work supports the notion that the oncogenic role of ABL in GBM cells is associated with its capability to coordinate a signalling setting that determines tumorigenic and stem-cell like properties.

## INTRODUCTION

The non-receptor tyrosine kinase (RTK) ABL influences behaviour of cells by regulating migration, invasion, survival, and proliferation, according to the cellular context [1–3]. ABL was shown to regulate cell membrane protrusions by modulating actin polymerization [4] and to control cell polarity by acting on polarized junctional dynamics in drosophila embryos [5, 6]. Through these mechanisms, ABL influences cell motility and directional collective cell migration, processes occurring during embryogenesis and cancer. ABL also participates in molecular events regulating the epithelial-mesenchymal transition [7]. Genetic studies using knock-out mice have highlighted the developmental requirement of Abl in cardiac growth [8], hepatocyte survival [9], neurulation [10, 11], and together with its homologue Arg in basement membrane integrity and cortical lamination in the cerebellum [12]. Furthermore, Abl influences mouse female fertility during chemotherapy [13] and its alteration may impact neurodegenerative diseases and therapies [14].

The BCR-ABL fusion protein, generated following translocation of *ABL* to the *BCR* gene, leads to constitutive activation of the ABL tyrosine kinase in 95% of chronic myeloid leukemia and cells depend on BCR-ABL activity for the execution of the oncogenic program [15]. In solid tumours, ABL is constitutively activated in breast carcinomas [16], non-small cell lung carcinomas [17], melanoma [18], anaplastic thyroid cancers [19], hepatocellular [20], ovarian [21], and gastric carcinomas [20]. In these tumours, ABL alterations occur through mechanisms distinct from gene mutation/translocation [1, 3, 22, 23]. For example, deregulated ABL is found in cancer cells with aberrant activation of RTKs, such as PDFGR, FGFR, EGFR, MET, KIT, and IGF1R [1, 3, 22]. In this context, a number of apparently contradicting results have shown that ABL acts as a signalling promoter [16, 18, 20, 24–27] or as a signalling inhibitor [28–31] of a given biological response, thus functioning as a “switch modulator”. These opposing effects are most likely related to how ABL is integrated into the oncogenic signalling machinery operating in cells. We have previously demonstrated that ABL acts as a signalling node interconnecting RTK and p53 “core pathways” during embryogenesis [9] and in cancer [20]. The implication of ABL in regulating the biology of cancer cells and the availability of clinically-relevant ABL antagonists has fostered exploration of their use in preclinical models and in clinics [1, 3, 22]. Most promising agents include Imatinib (Gleevec, STI571), Nilotinib, and Dasatinib. However, action of these antagonists is not restricted to ABL inhibition: Imatinib blocks PDGFR, KIT, ABL and its homologue ARG at comparable concentration levels [32]. Nilotinib is a second generation inhibitor that blocks preferentially ABL/ARG than PDGFR and KIT [32]. Dasatinib, a dual SRC/ABL inhibitor that also targets EGFR and KIT, elicits anti-tumorigenic effects in preclinical studies [33, 34]. While ABL antagonists are effective in clinics for CML treatment [35], their failure or limited success on solid tumours left open the debate as to whether they are ineffective or whether they must be used on patient subgroups characterised by a specific molecular signature. Furthermore, the use of ABL antagonists in combination with other agents for synergistic treatments remains an attractive possibility, although challenged by the limitless possibilities of drug combinations [3].

Glioblastoma multiforme (GBM), the most common and aggressive primary brain tumour in adults, can develop de novo (primary GBM) or through malignant progression of a low grade astrocytoma (secondary GBM) [36]. Patients suffering of GBM have a poor prognosis with a median survival rate of 12-15 months despite heavy clinical management including surgical ablation combined with Temozolomide chemotherapy and radiotherapy [37, 38]. Limited response to current GBM therapies is attributed to the presence of cells with stem-cell like properties, the so-called cancer stem cells [39–41]. These cells display the characteristic features of unlimited growth, self-renewal, differentiation, and are thought to be responsible for initiation, maintenance, and recurrence of tumours [42, 43]. A systematic analysis of (epi)genetic alterations in GBM led to the discovery of three main “core pathways” that are concomitantly altered: RTK signalling, p53, and RB “core pathways” [44]. The identification of altered molecular components through this and other GBM genome studies has boosted cellular and preclinical exploration of targeted molecular therapies to treat GBM. Relevance of RTKs in GBM is further supported by the constitutive expression of distinct RTKs that renders cells resistant to treatment with a single RTK blocking agent, a mechanism known as “RTK swapping” [45, 46]. For example, in a subgroup of GBM cells with constitutive activation of PDGFR, EGFR, and MET, the combination of Imatinib, SU11274, and Gefitinib elicits maximal response of GBM cells to treatment [45]. In GBMs with aberrant RTKs, ABL has been reported to be activated by and required for PDGFR function [47]. Reverse phase protein lysate arrays on high-grade *versus* lower-grade gliomas have identified ABL among the 12 most powerful discriminators [48]. Focal accumulation of ABL protein was also detected by immunolabelling in a proportion of GBM patients [49]. However, the extent to which ABL signalling influences GBM cell biology still remains elusive as: a) ABL and PDGFR reciprocally regulate their phosphorylation levels in GBM cells [47]; b) the beneficial effects of Imatinib cannot be attributed to one single target, since it simultaneously inhibits PDGFR and ABL (in addition to KIT) [45, 50]. In the present study, we used GBM as a cellular paradigm to explore whether and how permanent impairment of ABL function impacts biological and tumorigenic properties.

## RESULTS

### ABL inhibition leads to morphological and molecular changes in GBM cells

**Figure 1:**
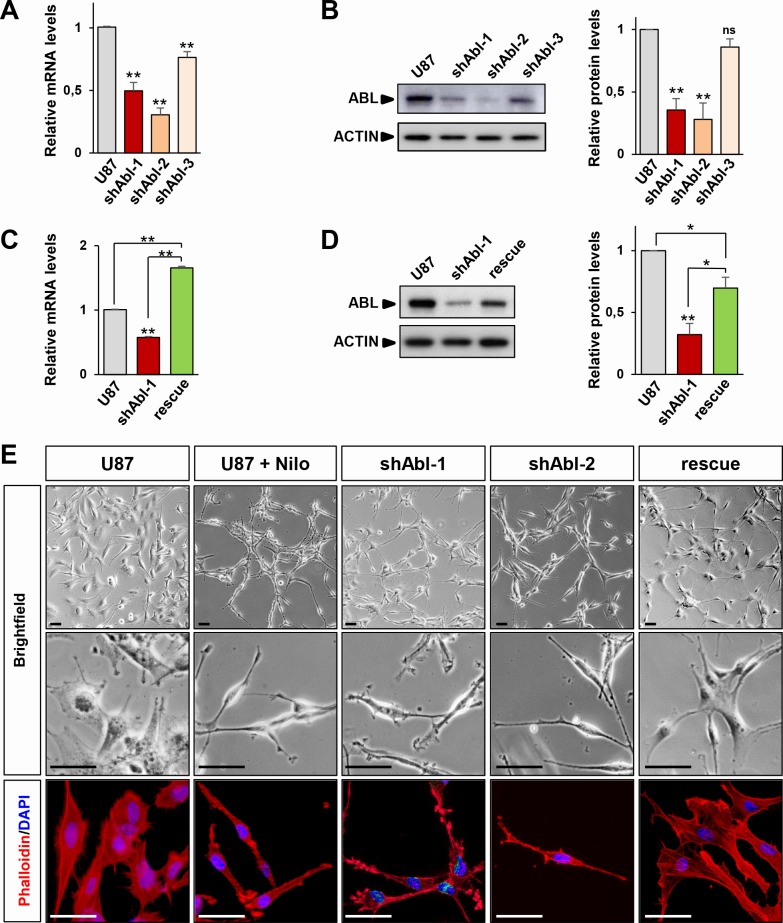
ABL impairment confers mesenchymal morphology of U87 cells **A**, **B.** RT-qPCR (**A)** and western blot (**B)** analyses of ABL expression levels in U87^shABL^ cells (U87 transfected with shABL-1, shABL-2, and shABL-3) compared to controls. Note ABL down-regulation in U87^shABL^ cells *versus* controls (for shABL-1: mRNA 0.5±0.06 fold change; protein: 0.35±0.09 fold change; for shABL-2: mRNA 0.3±0.05 fold change; protein: 0.28±0.13 fold change; for shABL-3: mRNA 0.76±0.04 fold change; protein: 0.86±0.06 fold change). **C.**, **D.** RT-qPCR (**C)** and western blot (**D)** analyses of ABL expression levels in U87^rescue^ compared to U87^shABL^ (U87 transfected with shABL-1) cells and controls. Note increased ABL levels in U87^rescue^ *versus* U87^shABL^ cells (mRNA: 1.65±0.03 fold increase; protein: 0.69±0.08 fold increase). The housekeeping gene *Beta-2-microglobuline* (*B2M*) was used as internal control in all RT-qPCR analyses, and ACTIN as loading control in all western blots. Results are the mean of at least three independent experiments. Values are expressed as means ± s.e.m. ns: not significant; * *P* < 0.05; ** *P* < 0.01. **E.** Brightfield (top and middle) and phalloidin staining (red; bottom) images of untreated U87, U87 cells exposed to Nilotinib (2μM; for 72 hrs), U87^shABL^ (shABL-1 and shABL-2), and U87^rescue^ cells. Note that ABL inhibition leads to the acquisition of a fusiform cell shape whereas U87^rescue^ cells exhibit a flatter phenotype. Nuclei stained with DAPI are in blue. Scale bars: 50μm.

The U87 GBM cell line has been extensively used to assess the effectiveness of several drugs targeting signalling components such as RTK inhibitors [45, 51–54]. We therefore used U87 as a GBM cell paradigm to assess the molecular and cellular consequences of ABL inhibition. We interfered ABL functions by two complementary approaches, involving either short hairpin RNA (shRNA) interference or pharmaceutical inhibitors. To achieve successful ABL targeting with the shRNA strategy, the efficiency of 3 different shRNA sequences to down-regulate ABL was tested in transfected cells after selection (Supplementary Table 1). Efficient downregulation of ABL, but not ARG, mRNA and protein levels was obtained by shAbl-1 [20, 55], shAbl-2, and to a lesser extent by shAbl-3 compared to either parental cells or cells transfected with a scrambled shRNA (both conditions further designed as control U87 cells; Figure 1A, 1B, and Supplementary Figure 1A). To reinforce specificity of the shRNA sequences used for targeting ABL, U87 cells carrying the shABL-1 plasmid were transfected with a vector expressing wild-type ABL. Increased ABL mRNA and protein levels were observed in these cells (Figure 1C and 1D). Through these studies, U87^shABL^ refer to stable ABL-mutant cells and U87^rescue^ to U87^shABL^ cells transfected with a vector expressing wild-type ABL. Concerning the pharmacological approach, ABL activity assessed by following its phosphorylation on tyrosine residues was impaired using Nilotinib, known to preferentially block ABL at 1-5μM (Supplementary Figure 1B). Intriguingly, we found that U87^shABL^ cells adopted a striking morphological change compared to controls, with fewer sites of cell contacts, and a major reorganization of actin filament distribution consistent with a loss of tight junction features (Figure 1E and Supplementary Figure 2A). Consistently, Nilotinib treatment of U87 cells led to a similar phenotype (Figure 1E) and did not further modify the morphology of U87^shABL^ cells (Supplementary Figure 1C). Restored ABL levels converted the spindle shape morphology of U87^shABL^ cells into a flatter appearance with increased cell contacts in U87^rescue^ cells (Figure 1E).

**Figure 2:**
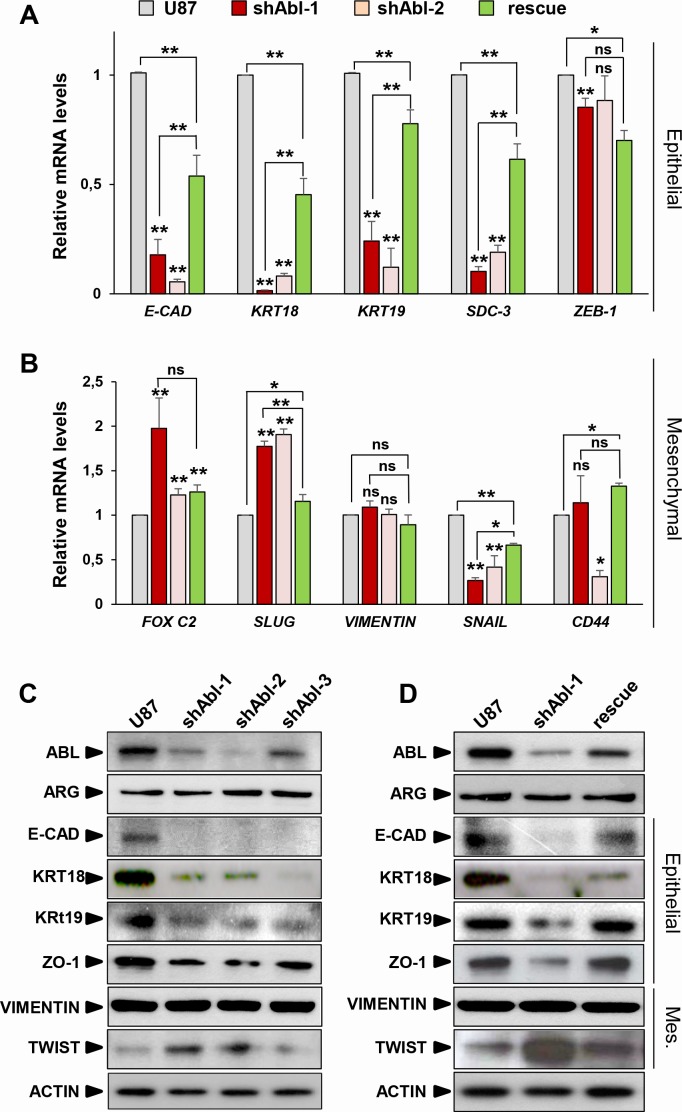
ABL impairment increases mesenchymal molecular traits at the expense of epithelial markers (A, B) RT-qPCR analysis showing repression of the epithelial markers (**A**), such as *E-Cadherin* (*E-CAD*), *Cytokeratin-18* (*KRT18*), *Cytokeratin-19* (*KRT19*), *Syndecan-3 (SDC-3)*, and *ZEB-1*. Concerning mesenchymal markers (**B**), *FOX C2* and *SLUG*, but not *VIMENTIN*, are up-regulated in U87^shABL^ cells compared to controls. Note down-regulation of *SNAIL* levels in U87^shABL^ cells. Levels of *CD44*, which is considered as a marker of mesenchymal or stemness traits depending on the culture conditions, are also reported. Quantitative analysis in U87^rescue^ cells shows significant increase of all epithelial markers accompanied by a decreased expression of *FOX C2* and *SLUG* mesenchymal markers. **C**, **D.** Western blots showing decrease in the expression levels of E-Cadherin (E-CAD), Cytokeratin-18 (KRT18), Cytokeratin-19 (KRT19), Zonal Occludens-1 (ZO-1), and increase of TWIST-1 (TWIST) in U87^shABL^ compared to controls (**C**). Restoration of E-CAD, KRT18 (although only partial), KRT19, and ZO-1 levels together with a decrease in TWIST levels were observed in U87^rescue^ cells (**D**). Mes: Mesenchymal. Results are the mean of three independent experiments. Values are expressed as means ± s.e.m. ns: not significant; * *P* < 0.05; ** *P* < 0.01.

Although U87 cells exhibit an epithelial morphology (as assessed by ATCC), they express both mesenchymal and epithelial markers. We therefore asked whether the phenotypic modifications caused by ABL inhibition in U87^shABL^ cells were accompanied by changes in the expression levels of epithelial and mesenchymal-related genes. U87^shABL^ cells undergo a strong repression of several epithelial markers, such as *E-Cadherin*, *Syndecan-3,* Zonal Occludens-1, Cytokeratin-18, and Cytokeratin-19, coinciding with changes in mesenchymal markers: *FOX C2, SLUG,* TWIST-1 were up-regulated, and *SNAILl* was down-regulated (Figure 2A-2D and Supplementary Figure 2B and 2C). Expression levels of epithelial and mesenchymal markers were significantly restored in U87^rescue^ cells (Figure 2A, 2B, and 2D).

To strengthen these results, we next asked whether ABL inhibition could also cause similar morphological and molecular changes in other GBM cell lines with more epithelial characteristics (Supplementary Figure 4). We found that Nilotinib treatment of LN18 and LN229 GBM cells, in which ABL is expressed and constitutively activated, also resulted in the acquisition of a mesenchymal-like morphology (Supplementary Figure S4A and S4B) accompanied by changes in levels of some epithelial (*Cytokeratin-18*, *Syndecan-3, ZEB-1*) and mesenchymal (*SNAIL*, *CD44; VIMENTIN* as well for LN229 cells) markers (Supplementary Figure S4C-S4F). Thus, ABL impairment in GBM cells enhances both morphological and molecular mesenchymal characteristics.

### Increased migration and invasive properties of GBM cells with ABL impairment

**Figure 3:**
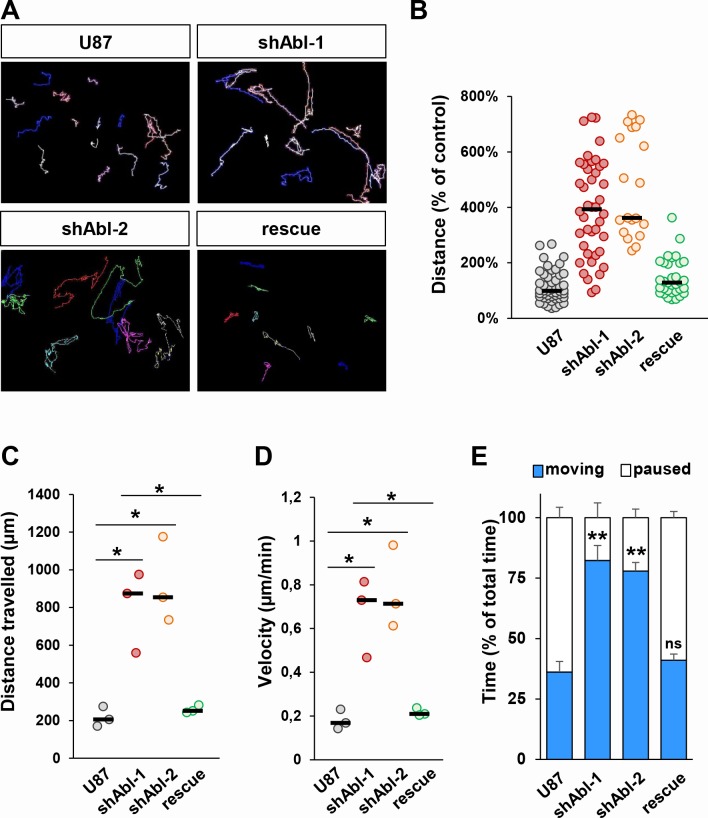
Increased migration properties of U87 cells with impaired ABL **A.** Representative images of migration paths of U87, U87^shABL^, and U87^rescue^ cells analysed by time-lapse videomicroscopy (*n* = 3). **B**-**E.** Quantification of time-lapse videomicroscopy showing the distance the cells travelled expressed as percentage of the distance travelled by control cells (**B**, each dot represents a single cell analysed), the total distance the cells travelled (**C**, each dot represents the mean of independent experiments; U87: 206.4μm±30.5μm; shABL-1: 874.1μm±125.3μm; shABL-2: 854.2μm±131.7μm; rescue: 251.7μm±12.1μm), the mean velocity of the cells during the 20hrs of recording (**D**, each dot represents the mean of independent experiments; U87: 0.17±0.02μm/min; shABL-1: 0.73±0.1μm/min; shABL-2: 0.71±0.11μm/min; rescue: 0.21±0.01μm/min), and the time percentage the cells spent moving *versus* paused (**E**). Values are expressed as means ± s.e.m. ns: not significant; * *P* < 0.05; ** *P* < 0.01.

Since ABL knockdown exacerbates the mesenchymal phenotype of U87 cells, we next investigated whether ABL inhibition influences cell motility and invasion. For cell motility, through time-lapse videomicroscopy we recorded several parameters: distance, velocity, path of migration, and the duration of motile *versus* paused behaviour. The migration capacity of U87^shABL^ cells was significantly enhanced, with about a 4-fold increase in the average distance travelled and in their mean velocity (Figure 3A-3D and Supplementary Figure 5A-5D). Moreover, whereas control cells paused for 65% of the time, U87^shABL^ cells spent about 80% of the time being motile (Figure 3E and Supplementary Figure 5E). The migration capacity of U87^rescue^ cells was drastically reduced compared to that of U87^shABL^ cells (Figure 3A-3E). These studies were performed in the presence of AraC in order to carefully analyse cell motility properties during 20 hrs, independently of cell division. However, as AraC can influence cell survival [56], motility studies were also done in the absence of AraC and led to the same results (Supplementary Figure 6A, 6B). Finally, using Matrigel-Boyden chambers to study the cell's invasive capacity, we found that invasiveness of U87^shABL^ cells was increased as compared to that of controls (Supplementary Figure 5F). Taken together, these findings show that the morphological changes caused by ABL impairment in GBM cells are accompanied by enhanced migration and invasion properties.

### ABL impairment leads to reduced proliferation and drastically impacts the tumorigenic properties of GBM cells both *in vitro* and *in vivo*

**Figure 4:**
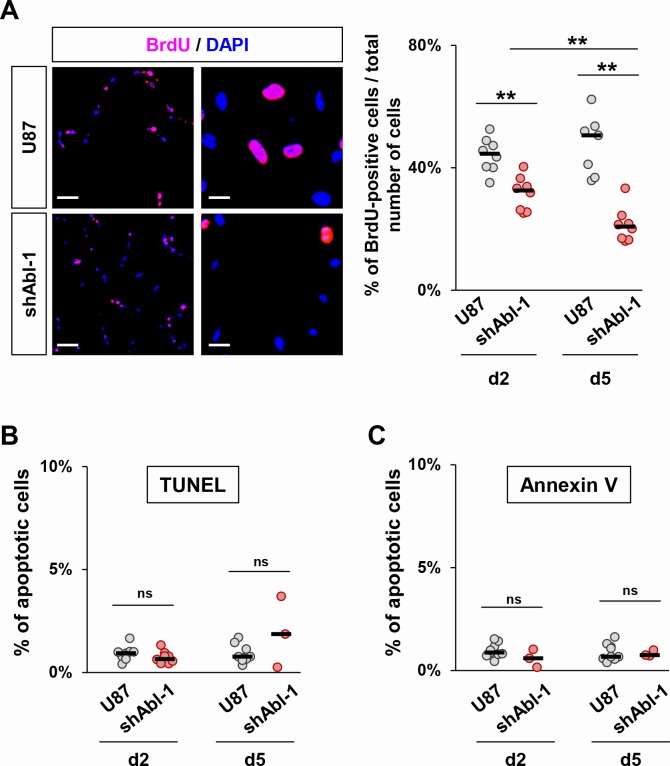
ABL inhibition decreases cell proliferation without affecting cell survival **A.** Representative images (left) and quantification (right) of cell proliferation determined by analysis of BrdU incorporation. The proliferation rate of U87^shABL^ cells is reduced compared to control cells (at day2, U87:44.2±1.9%; U87^shABL^:31.6±2.25%; at day5, U87:47.5±3.7%; U87^shABL^:21.3±2%). Scale bars correspond to 250μm (left) and 50μm (right). **B**, **C.** Quantitative analysis of apoptotic cells as assessed by either TUNEL (**B**) or Annexin V expression (**C**) in U87 and U87^shABL^ cells. Each dot represents the mean of independent experiments. ns: not significant; ** *P* < 0.01.

**Figure 5:**
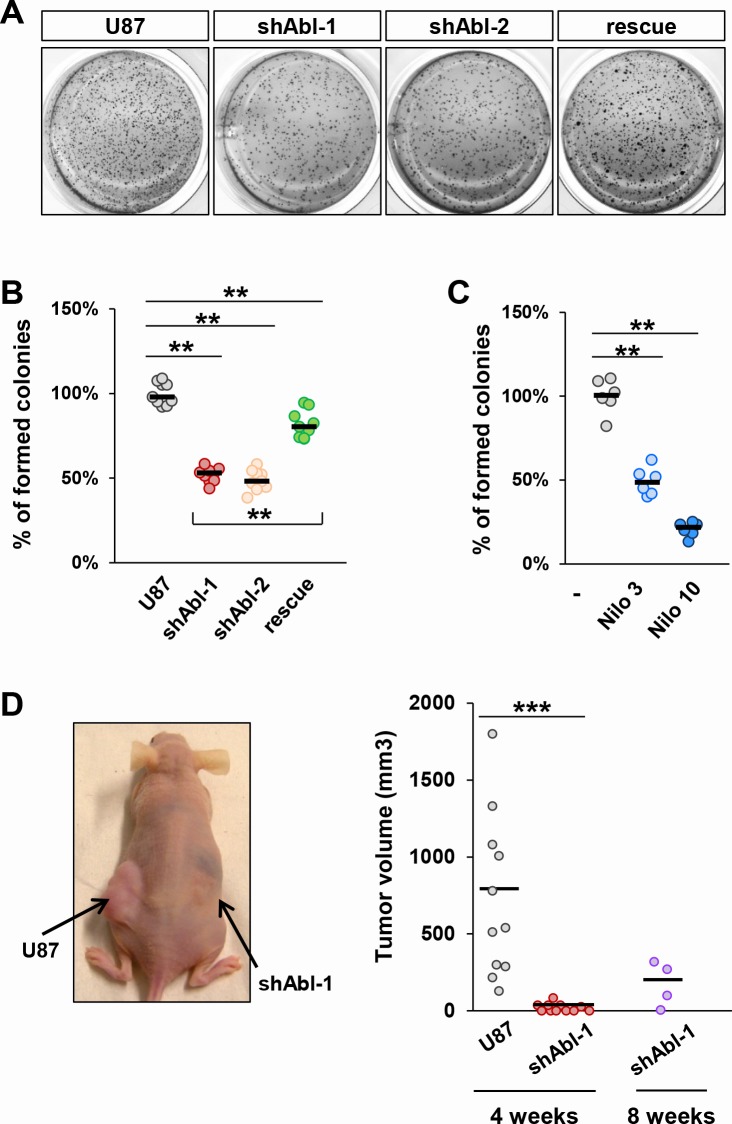
Tumorigenic properties of U87 cells are impaired by ABL inactivation both *in vitro* and *in vivo* **A**-**C.** Anchorage-independent growth assay showing reduced *in vitro* tumorigenic properties of U87^shABL^ (shABL-1: 52.05±2.1%; shABL-2: 48.9±2.2%) and partial restoration in U87^rescue^ cells (**A** and **B**; 81.8±2.3%;). Reduced tumorigenic properties of Nilotinib-treated U87 cells compared to controls (**C**; 3μM: 49.2±3.3%; 10μM: 20.7±1.7%). Each dot represents the mean of independent experiments. **D.** Xenograft studies were performed by subcutaneous injection of U87 (left flank) and U87^shABL^ (right flank) cells. A representative mouse 4 weeks after injection is shown (left panel). Quantitative analysis of the volume of dissected tumours 4 and 8 weeks after cell injection (right panel). Note that after 4 weeks, U87 cells formed in all injected mice tumours of an average volume of ~720 mm^3^, whereas U87^shABL^ cells exhibited a drastically reduced capacity to form tumours (mean tumour volume: ~20mm^3^), which were detected in only 54% of injected mice. After 8 weeks, the mean of tumour volume generated by U87^shABL^ cells was ~170 mm^3^. Each dot corresponds to the tumour value of one mouse. ** *P* < 0.01; *** *P* < 0.001.

We next investigated whether permanent ABL impairment impacts the cell proliferation capacity by following BrdU incorporation. Quantification analysis revealed a decrease in the percentage of BrdU-positive U87^shABL^ cells compared to controls (Figure 4A). In contrast, no changes were observed in the proportion of apoptotic U87^shABL^ and control cells (Figure 4B and 4C). These results prompted us to assess whether and to what extent ABL inhibition could compromise the tumorigenicity of GBM cells. We performed soft-agar assays to evaluate the cell's ability to grow in an anchorage-independent manner, a hallmark of cancer cells. We found about 50% decrease in colony number formed by U87^shABL^ cells compared to controls (Figure 5A, 5B, and Supplementary Figure 7A). Tumorigenic capacity was significantly restored in U87^rescue^ cells (Figure 5A and 5B). Pharmacological inhibition of ABL impacts tumorigenicity of U87, as well as LN18 and LN229 cells, in a dose-dependent manner (Figure 5C and Supplementary Figure 7B and 7C), pointing to a requirement for ABL in the oncogenic program of GBM cells we tested. We next performed xenograft studies and found that U87^shABL^ cells fail to develop tumour masses in contrast to controls (Figure 5D). Together, these results provide evidence that intact ABL confers tumorigenicity to GBM cells.

### ABL knock-down affects self-renewal capability of GBM cells

**Figure 6:**
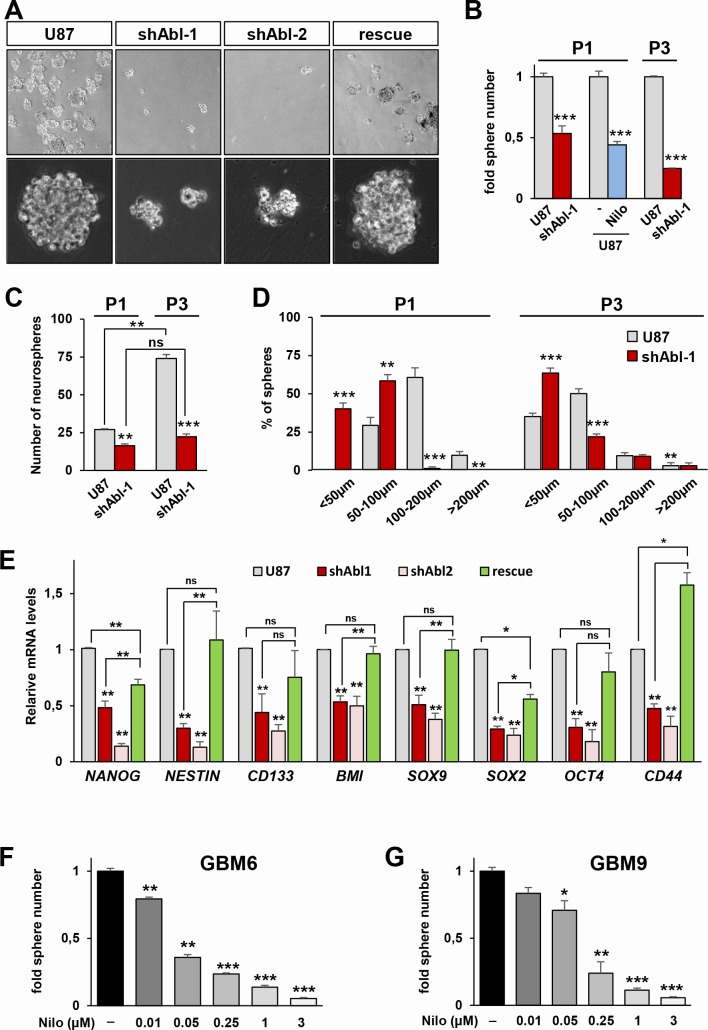
Permanent ABL inhibition interferes with neurosphere formation and expression of self-renewal markers in GBM cells **A.** Representative images of spheres derived from U87, U87^shABL^, and U87^rescue^ cells. **B, C.** Reduced numbers of spheres derived from U87^shABL^ *versus* U87 cells at passage 1 (P1) and 3 (P3). Nilotinib treatment (Nilo; 3μM) also reduces the number of U87 spheres at P1. In B, results are expressed as fold changes in sphere numbers between cells with ABL inhibition *versus* controls. In C, results are expressed in terms of number of formed spheres compared to the number of seeded cells. This way of reporting data determines the sphere forming efficiency of each cell line, emphasizing differences in self-renewal capacity. Note that as the number of passages increases (from P1 to P3), the sphere forming efficiency of U87 cells raises up (2.7 fold), whereas the capacity of U87^shABL^ cells does not significantly change. **D.** Size of spheres derived from U87 and U87^shABL^ cells analysed after P1 and P3. **E.** RT-qPCR analyses showing the repression of pluripotent markers (*NANOG, NESTIN, CD133, BMI, SOX9, SOX2, OCT4*, and *CD44*) in U87^shABL^ compared to U87 spheres. Partial restoration of most of the stemness markers was observed in U87^rescue^ cells. Data correspond to biological triplicates. **F**, **G.** GBM6 (**F**) and GBM9 (**G**) cells in neurosphere conditions were exposed to various doses of Nilotinib (μM). Sphere numbers were quantified after 14 days of culture. Each experiment was done in triplicate. Values are expressed as means ± s.e.m. ns: not significant; * *P* < 0.05; ** *P* < 0.01; *** *P* < 0.001.

We next asked whether the loss of tumorigenicity of U87^shABL^ cells is at least partially due to a modification in their tumour initiating properties. Pseudo-sphere formation assays (also called neurosphere assays) were performed in stem cell-permissive media to examine the U87 cell self-renewal capacity [57]. We found that the total number of spheres derived from U87^shABL^ cells is significantly reduced compared to controls (Figure 6A-6C). Similar results were observed by pharmacological ABL inhibition with Nilotinib (Figure 6B). The self-renewal capability of U87^shABL^ cells was next examined after dissociation of primary spheres and low-density replating to form secondary spheres. At passage 3, the overall number of spheres generated from U87^shABL^ cells is more than three-fold reduced compared to controls (Figure 6B-6D). Since the same number of cells were seeded in all conditions, we were able to assess the sphere forming efficiency of both cell lines overtime. Data show that, as the number of passages increases (from P1 to P3), the sphere forming efficiency of U87 cells raises up (2.7 fold), whereas the capacity of U87^shABL^ cells does not significantly change (Figure 6C). Moreover, a detailed analysis at passage 1 and 3 showed that the majority of spheres derived from U87^shABL^ cells are smaller than 100μm (Figure 6D).

To identify the molecular changes underlying the reduced self-renewal capacity resulting from ABL impairment, we analysed the expression levels of several stem cell markers and found that *NANOG, NESTIN, CD133, BMI, SOX9, SOX2, OCT4*, and *CD44* (considered to be a marker of mesenchymal stem cells) were significantly downregulated in spheres derived from U87^shABL^ cells compared to controls (Figure 6E and Supplementary Figure 8B). Restoration of ABL levels led to a raise in number and size of spheres as well as expression levels of most stem cell markers in U87^rescue^ spheres (Figure 6A and 6E). Altogether, these results indicate that permanent ABL inhibition interferes with the expression of stemness markers and with sphere formation capacity of U87 cells.

We further investigated whether ABL targeting also affects stem cell-like properties in human primary GBM cells. We used GBM6 and GBM9 stem-like cells with different molecular and biological properties, capable of self-renewing and generating infiltrative tumours after grafting into nude mice [58, 59]. Nilotinib treatment prevented the GBM neurosphere formation in a dose-dependent manner (Figure 6F and 6G). Notably, Nilotinib impairs self-renewal capacity of GBM6 and GBM9 cells at lower doses as compared to U87 cells (around 100-fold less), a remarkable effect considering that stem cells, thought to be responsible of tumour recurrence, are known to be resistant to several drugs. Collectively, these results highlight that intact ABL is required to maintain stem-cell like properties in the GBM cells we tested.

### Permanent ABL inhibition causes a drastic change in the signalling network components of GBM cells

**Figure 7:**
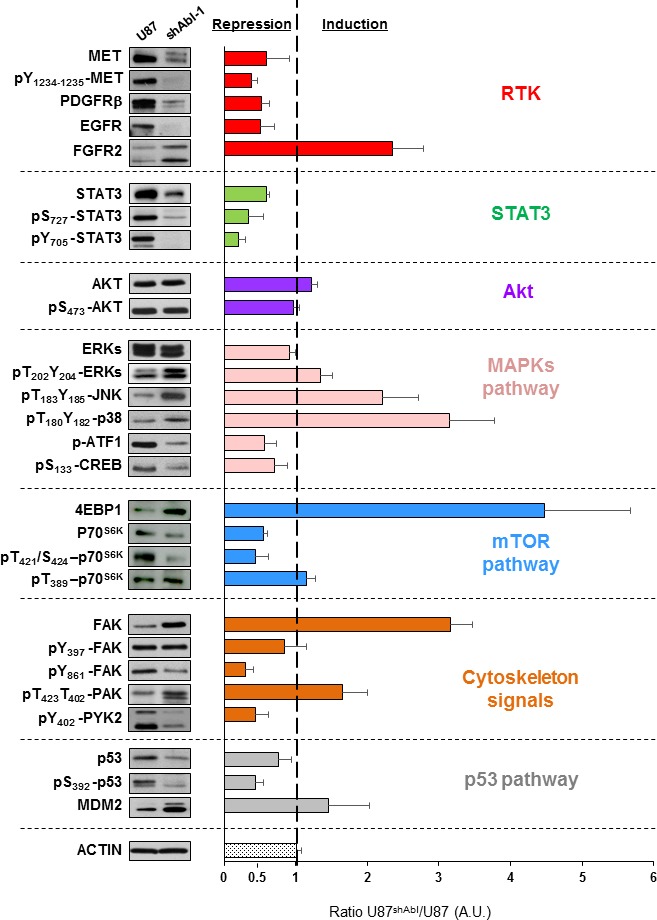
ABL down-regulation modifies multiple signalling components in GBM cells Western blots showing the expression and phosphorylation levels of RTKs (MET, pY_1234-1235_-MET, PDGFRβ, EGFR, FGFR2), AKT (AKT, pS_473_-AKT), STAT3 (STAT3, pS_727_-STAT3, pY_705_-STAT3), MAPKs pathway (ERKs, pT_202_Y_204_-ERKs, pT_183_Y_185_-JNK, pT_180_Y_182_-p38, p-ATF1, pS_133_CREB), mTOR pathway (p70^S6K^, pT_421_S_424_-p70^S6K^, pT_389_-p70^S6K^, 4EBP1), cytoskeleton signals (FAK, pY_397_-FAK, pY_861_-FAK, pT_423_T_402_-PAK, pY_402_-PYK2) and p53 pathway (p53, pS_392_-p53, MDM2) in U87 and U87^shABL^ cells. Representative western blots are shown on the left. The graph on the right shows the ratio of expression or phosphorylation levels of the indicated proteins in U87^shABL^ *versus* U87 cells (quantification from three independent experiments). A.U: Arbitrary units. Values are expressed as means ± s.e.m.

**Figure 8:**
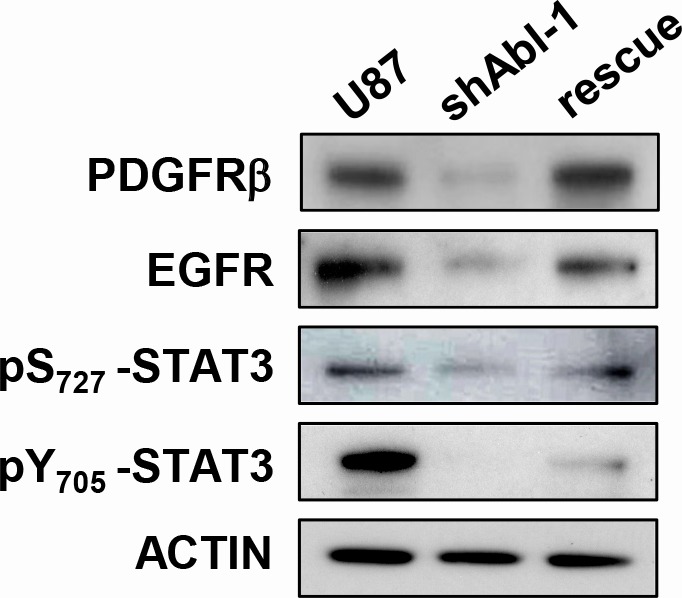
Signalling status in GBM cells is sensitive to ABL threshold Western blots showing expression levels of PDGFRβ and EGFR as well as the phosphorylation status of pS_727_-STAT3, pY_705_-STAT3 in U87, U87^shABL^, and U87^rescue^ cells.

The severe impact on the tumorigenic and self-renewal properties of GBM cells with impaired ABL function can be interpreted at least in two ways. One scenario could be that ABL participates in the oncogenic program by ensuring the activation of one key signalling component (or a small number of them): its inhibition would therefore interfere with the oncogenic execution of upstream regulators by altering a key modulator. Alternatively, ABL might act as a coordinator of the overall signalling threshold: its inhibition would therefore impact the oncogenic program as the signalling machinery is disrupted at multiple points. To discriminate between these two possibilities, we analysed the expression and phosphorylation levels of several signals known to regulate the tumorigenic and self-renewal properties of GBM cells. Remarkably, we found a drastic alteration of multiple RTKs and intracellular signals in U87^shABL^ cells compared to controls. These changes include: a) loss of MET, PDGFRβ and EGFR expression, and of MET phosphorylation; b) down-regulation of the phosphorylation levels of STAT3, ATF-1, CREB, and p70^S6K^; c) changes in p53 expression and phosphorylation on Ser_392_ that confers transcriptional competence towards specific targets [9, 20] (Figure 7). Interestingly, expression and phosphorylation levels of some signalling molecules, such as PDGFRβ, EGFR, pS_727_-STAT3, and pY_705_-STAT3, are either totally or partially restored in U87^rescue^ cells (Figure 8). Together, these results indicate that ABL acts as a signalling coordinator by ensuring the expression and/or phosphorylation levels of multiple components known to participate to the tumorigenic properties of GBM cells.

## DISCUSSION

**Figure 9:**
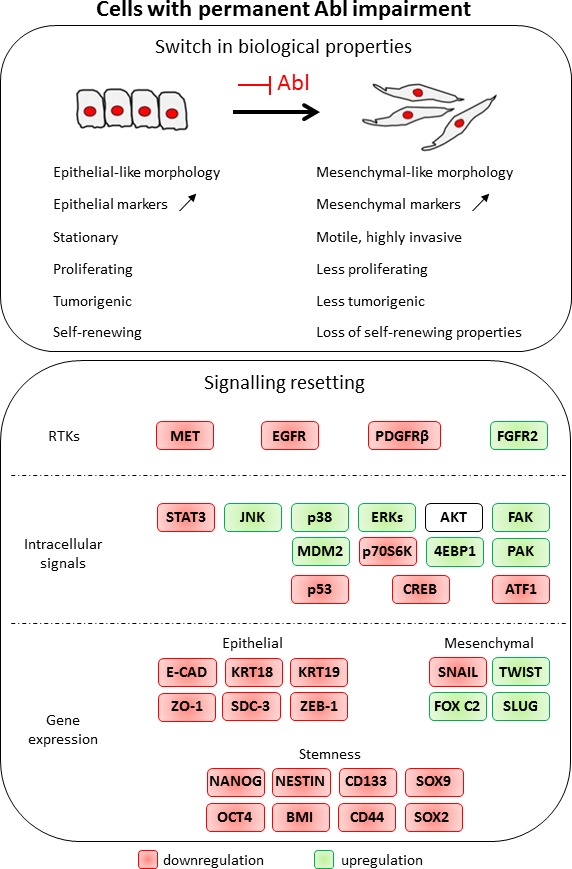
Permanent Abl ablation in GBM cells leads to a switch in biological properties and to a signalling resetting

The effect of Imatinib on cancer cells including GBM has been mainly attributed to PDGFR inhibition [45, 50, 60], although it also targets ABL and KIT. Interpreting the Imatinib effects on GBM cells is further confounded by the existence of a regulatory feedback loop in which ABL and PDGFR reciprocally regulate their phosphorylation levels [47]. Our studies highlight that permanent ABL inhibition in GBM cells leads to profound changes at molecular and biological levels: the mesenchymal features of GBM cells are exacerbated as shown by loss of epithelial-like polarity, increased migration and invasion capacity, whereas proliferation and tumorigenesis are compromised (Figure 9). Our studies uncover an additional feature of ABL only reported so far in CML: the regulation of stem-cell like properties. This is supported by our observation that ABL inhibition down-regulates expression of stem-cell markers in sphere-cultured conditions and prevents neurosphere formation (Figure 9). Collectively, these studies support the notion that the oncogenic role of ABL in solid tumours, like GBM, relies on its capability to coordinate a signalling setting that determines cell tumorigenicity and stem-cell like properties.

### ABL coordinates multiple biological properties in GBM cells

The present study extends previous reports on persistent ABL inhibition in cancer cells [61] and reveals dramatic molecular and behavioural consequences of permanent ABL impairment overtime. The enhanced mesenchymal traits conferred by ABL targeting in GBM cells impact their biological properties as illustrated by increased migration/invasion. This is consistent with molecular changes occurring in cells with impaired ABL: down-regulation of epithelial markers (such as *E-Cadherin, ZO-1, Cytokeratin-18, Cytokeratin-19, Syndecan-3,* and *ZEB-1* to a lesser extent) and up-regulation of mesenchymal markers (such as *FOX C2, SLUG,* and *TWIST-1*). The acquisition of molecular and morphological traits by targeting ABL appears to be a dynamical process as some changes are already observed within 48 hrs of ABL inhibition (Supplementary Figure 9A-9C), which is further supported by the reversion of these traits when ABL expression levels are restored. The competence of ABL to influence epithelial/mesenchymal features is corroborated by the acquisition of mesenchymal-like morphology of epithelial-like GBM cells such as LN18 and LN229. Remarkably, the proliferation and the tumorigenicity of GBM cells are compromised when ABL is impaired. In contrast to our findings, it has been shown that increased expression and activation levels of ABL and ARG lead to enhanced motility and invasive properties of breast cancer cells [1, 3, 16, 62, 63]. This is coherent with their engagement in the actin polymerisation machinery, which leads to the formation of membrane protrusions, morphological changes, and affects cell adhesion and migration [23]. Among putative signals conferring enhanced motility in GBM cells with permanent ABL targeting, ARG could be implicated also taking into account its reported localisation in invadopodia (actin-rich structures) and its involvement in extracellular matrix degradation and invasion [63, 64]. Nevertheless, the overall signalling resetting we report in cells with permanent ABL impairment could underlie the involvement of other regulators of cell motility/invasion.

Changes in GBM cell behaviour are most likely caused by the drastic alterations of signalling components we highlighted. Permanent ABL impairment leads to down-regulation in the levels of PDGFR/EGFR expression as well as of MET expression/phosphorylation, reinforcing the existence of a feedback loop between ABL and RTKs [26, 47]. Such down-regulation of RTKs could be per se incompatible with the tumorigenic competence of GBM cells [45, 65–67]. Consistently, drugs targeting several RTKs at the same time elicit efficient responses on a range of GBM cells [45, 50, 54]. Besides RTKs, we show a severe alteration also of MAPKs, STAT3, and p53 intracellular pathways, each of them being able to contribute to GBM tumorigenicity [68, 69, 70]. In our opinion, such signalling resetting likely reflects direct effects of reduced ABL inputs for some signals (known as ABL effectors) and indirect consequences of ABL impairment overtime for others. Collectively, this resetting implies that ABL acts as a signalling coordinator by (directly or indirectly) ensuring the expression and/or phosphorylation levels of multiple components known to participate to the tumorigenic properties of GBM cells. An intriguing question we also addressed is to which extent the overall changes occurring in cells with ABL impairment are reversible. The U87^rescue^ cells with restored ABL levels have been instrumental not only to rule out off-target effects of the shRNA approach, but also to demonstrate that signalling, molecular, and biological features of ABL-targeted cells are reversible. Such reversibility underlines a remarkable plasticity of GBM cells to ABL threshold.

### Uncoupling mesenchymal from stem-cell like features in GBM cells by targeting ABL

Cancer cells can de-differentiate through aberrant activation of epithelial-mesenchymal transition thereby increasing cancer cell motility and dissemination, but also promoting their self-renewal capability [71]. The direct link between mesenchymal features and stemness has been supported by several studies showing that activators of the epithelial-mesenchymal transition (such as TWIST1, SNAIL1, SLUG, ZEB1) also confer stemness properties [72–74]. However, an exception to this widely accepted concept has been reported. It is the case of PRRX1, an activator of epithelial-mesenchymal transition that nevertheless suppresses stemness traits [75]. In particular, PRRX is coexpressed and cooperates with TWIST1 in favouring cell migration and invasion while depleting stemness properties [75]. The biological and mechanistic outcomes from these studies highlight the possibility of uncoupling stemness from mesenchymal traits in cancer cells [76]. Such a new concept has recently been supported by an elegant study based on a breast model of tumorigenesis in which epithelial-mesenchymal regulators have not been genetically modified. The authors have shown that the epithelial-mesenchymal plasticity occurring in metastatic process appears to be irrelevant for differential stemness capacity [77]. Our studies provide an additional example of uncoupling mesenchymal and stem-like features. Indeed, the enhanced mesenchymal properties in GBM cells with ABL ablation is paralleled by the loss of stem cell markers, such as *NANOG*, *NESTIN*, *CD133*, *BMI*, *SOX9, SOX2*, and *OCT4*, and reduced neurosphere formation. Moreover, our cellular system demonstrates a remarkable plasticity of epithelial/mesenchymal *versus* stemness traits according to ABL thresholds (by comparing U87, U87^shAbl^, and U87^rescue^ cells). To our knowledge, this is the first example linking ABL to stem-cell like properties in solid tumours, compatible with haematopoietic stem cell renewal by BCR-ABL in CML [78, 79]. Such modulation of stem cell-like properties may offer an additional mechanism to contrast GBM tumorigenicity in cells that make use of ABL to exacerbate stemness maintenance. Future studies will determine whether the uncoupling of mesenchymal and stem-cell like features with ABL inhibition also occurs in other cells derived from solid tumour, particularly those in which ABL has been reported to elicit functional responses. How ABL may impact stemness is an intriguing question that deserves several biochemical and molecular screens performed in a panel of cells. It is tempting to speculate that reduced stemness in GBM cells lacking ABL is likely due to the alteration of multiple components, including receptors of extracellular signals and intracellular effectors.

### Could ABL be a target for GBM therapies?

The drastic consequence of targeting ABL in GBM cells like those used in our study solicits the question of whether ABL antagonists can be relevant in therapy. This possibility is supported by our findings showing that permanent ABL ablation causes a dramatic change in the expression/activation levels of multiple signals relevant in GBM biology. Furthermore, as ABL inhibition impinges also on stemness properties of GBM cells, it will be important to assess whether cancer stem cells become more sensitive to chemotherapeutic agents or radiotherapy. Although some clinical trials have not shown any beneficial effects of targeting ABL for GBM treatment [48, 60, 80], additional trials using ABL inhibitors are currently ongoing (e.g.http://clinicaltrials.gov/show/NCT01140568; https://www.klinikum.uni-heidelberg.de/Recurrent-glioblastoma-WHO-grade-IV.108973.0.html?&L=%201). An easy conclusion from failure of completed trials is that while ABL antagonists are highly effective on CML cells addicted to the BCR-ABL oncogenic form, they elicit moderate response on cells in which the oncogenic contribution of not mutated ABL can be substituted by other signals. However, an alternative possibility is that we still do not know how to use ABL antagonists for GBM therapy. Examining beneficial (and limiting) effects of ABL inhibition for GBM treatment is conditioned by at least two main issues. First, the combined drug(s) to use for therapies is likely to determine the extent of success (or failure). However, determining which combinations maximize effectiveness among the limitless possibilities remains a major challenge. A strategy worth to explore could be to exacerbate the effects of ABL inhibition with agents eliciting cytotoxic effects [81, 82]. Second, the identification of a molecular signature would permit targeting of a GBM patient subgroup sensitive to ABL inhibitors. In this respect, previous studies identified candidate markers predicting the sensitivity of solid tumours to ABL inhibitors [20, 34, 83].

How can signatures of GBM patients sensitive to ABL antagonists be identified? It is tempting to speculate that ABL expression levels might not be the appropriate criteria to use, since ABL requirement is most likely determined by the oncogenic cascade operating in GBM. One possibility is to use a set of changes caused by ABL inhibition, such as those we highlight in this study, to define a “molecular code”. This code can then be used to search for putative patient subgroup(s) by bioinformatically revisiting GBM (epi)genomic databases. Such studies could also predict convergent and compensatory pathways in order to design optimal combined drug treatments. Nevertheless, we also propose to carefully take into account that the drastic molecular and biological effects of ABL inhibition in GBM cells are reversible, at least to a certain extent, once ABL threshold is restored. Addressing this issue will provide insights on whether constant treatment of GBM patients with ABL antagonists may be required to convert GBM malignancy into a stable chronic disease and/or whether Abl targeting therapies can only be effective when combined with cytotoxic agents.

## CONCLUSIONS

Overall our data support the concept that the oncogenic role of ABL in solid tumours relies on its capability to coordinate a signalling setting that determines tumorigenic and stem-cell like properties. Thus, our findings together with those from other studies reinforce the potential of treating solid tumours with ABL antagonists, most likely in combinatorial therapies. We believe that efficient ABL targeted therapies are conditioned by the identification of molecular signatures to delineate responding patients and by the assessment of whether ABL drug may convert a malignant into a stable chronic disease.

## MATERIALS AND METHODS

### Cell culture and compound treatment

Human U87-MG (U87), LN18, and LN229 cell lines were obtained from ATCC collection. The generation of human GBM6 and GBM9 cells derived from GBM patients has been previously described [58]. The U87 cell line was cultured in RPMI-1640 (Invitrogen Life Technologies) whereas the LN18 and LN229 cells were grown in Dulbecco's modified Eagle's media (DMEM; Invitrogen Life Technologies). Both media were supplemented with 10% (v/v) foetal bovine serum (FBS), 100U/mL penicillin, 100μg/mL streptomycin, 4mM L-glutamine, and 1mM of sodium pyruvate (defined as complete media). Cells were grown at 37°C in a humidified atmosphere of 5% CO_2_ and the medium was changed every 2-3 days. GBM6 and GBM9 cells were cultured in stem-cell permissive medium as previously reported [58]. Cells were cultured for 24h prior to Nilotinib treatment (Selleckchem; the time of treatment is indicated in figure legends).

### Cell transfection

For cell transfection, the following plasmids carrying shRNA sequences were used: pSUPER.retro with non-targeting shRNA sequence. pSUPER.retro with shABL-1 sequence (5′-AAAGGUGAAAAGCUCCGGGUC-3′) [20, 55]; pGIPz/puro with shABL-2 (5′- ATGCTTAGAGTGTTATCTC-3′) and shABL-3 (5′-AATGGAGCGTGGTGATGAG-3′) (shABL-2 and shABL-3 from Thermo Scientific). Note that the shAbl-3 targeting sequence is less efficient in downregulating ABL compared to shAbl-1 and shAbl-2. The moderate decrease of ABL levels in U87^shAbl-3^ cells is paralleled by a switch of some, but not all, molecular and cellular properties observed in U87^shAbl-1^ and U87^shAbl-2^ cells. Based on these findings, it is tempting to speculate that U87^shAbl-3^ cells underline a range of sensitivities to different ABL dosages. Below a certain threshold (modelled by U87^shAbl-3^ cells), ABL inputs are not permissive for some morphological, molecular, and biological properties. U87^rescue^ cells were generated by transfecting U87 cells carrying shAbl-1 with pSGT-ABL^wt^ plasmid (kindly provided by D. Barilà). Vectors were transfected into cells by using Lipofectamine 2000 (Invitrogen Life Technologies), according to the manufacturer's instructions. Cells were selected with G418 and pools of resistant cells were used for experiments.

### Total RNA extraction and quantitative real-time PCR

Total RNA was extracted from cells using the RNeasy mini kit (QIAGEN), processed with DNase (RNase-free DNase I Set, QIAGEN), and purified on RNAeasy column (QIAGEN). Reverse Transcription was then performed with iScript Reverse Transciption Supermix (Bio-Rad). mRNA levels were assessed in a qPCR CFX 96 apparatus (Bio-Rad). Amplifications were done using the SYBR^®^ Green detection method. The target mRNA levels were normalized to the housekeeping gene *Beta-2-microglobuline* (*B2M*) and were analysed using the 2^−ΔΔCt^ method. All reactions were run in triplicate and repeated in at least two independent experiments. The results are presented as n-fold changes *versus* the values in control cells. Primer sequences used are reported in Supplementary Table 2.

### Western blots and immunocytochemistry

For western blots, protein extracts were biochemically analysed as previously described [84]. Antibodies used were anti-Actin (1:1000), anti-Tubulin (1:4000; Sigma), anti-Vimentin (1:500; AbCam), anti-ABL (1:1000; Calbiochem), anti-Mdm2 (1:3000; Oncogene), anti-FAK (1:1000), anti-E-Cadherin (1:5000; BD Transduction Labs), anti-ZO-1 (1:2000; Invitrogen Life Technologies), anti-Met (1:1000; sc-161), anti-EGFR (1:1000; sc-03), anti-FGFR2 (1:1000; sc-122), anti-p53 (1:5000; sc-6243), anti-p70^S6K^ (1:1000; sc-230), anti-Cytokeratin 18 (1:100; sc-32329), anti-Cytokeratin 19 (1:200; sc-53003), anti-Twist (1:2000; H-81; Santa Cruz), anti-4EBP1 (1:500; 07-397; Upstate), anti-phosphotyrosine (1:1500; 4G10, Millipore), anti-phosphoY_397_-FAK (1:2000), anti-phosphoY_861_-FAK (1:1000), anti-phosphoY_402_-Pyk2 (1:2000; Biosource), anti-phosphoY_412_-ABL (1:1000), anti-phosphoY_1234-1235_-Met (1:2000), anti-PDGFRβ (1:1000), anti-Akt (1:2000), anti-phosphoS_473_-Akt (1:2000), anti-STAT3 (1:2000), anti-phosphoS_727_-STAT3 (1:2000), anti-phosphoY_705_-STAT3 (1:2000), anti-ERKs (1:10000), anti-phosphoT_202_Y_204_-ERKs (1:10000), anti-phosphoT_183_Y_185_-JNK (1:1000), anti-phosphoT_180_Y_182_-p38 (1:2000), anti-phosphoS_133_-CREB (1:1000; this antibody also recognizes p-ATF1), anti-phosphoT_423_T_402_-PAK (1:1000), anti-phosphoT_389_-p70^S6K^ (1:1000), anti-phosphoT_421_S_424_-p70^S6K^ (1:5000), anti-phosphoS_392_-p53 (1:1000; Cell Signaling). For screen studies of expression/phosphorylation levels, densitometric analysis was performed with the ImageJ software.

For immunocytochemistry, cells were cultured on coverslips, then fixed with 4% paraformaldehyde (PFA) for 10 minutes at room temperature. After permeabilization for 15 minutes with PBS-0.5% TritonX-100, cells were incubated for 1h with Alexa594-conjugated phalloidin (Life Technologies), then washed in PBS-0.1%TritonX100. Finally, coverslips were mounted in Prolong-Gold antifade reagent DAPI (Invitrogen Life Technologies).

### Time-lapse videomicroscopy

U87, U87^shABL^, and U87^rescue^ cells (1×10^4^) were seeded into 6 cm dishes and incubated under normal growth conditions for 24h. To avoid evaluating effects that would be secondary to changes in cell proliferation, all recordings were performed in presence of Cytosine arabinoside (AraC; 10μM; Sigma), an inhibitor of DNA synthesis. Experiments were also performed in the absence of AraC. The dynamics of cell movement at 37°C and 5% CO_2_ was monitored by time-lapse cinematography using an inverted optical microscope ObserverZ1 colibri1 (Zeiss) equipped with an incubator chamber placed on a motorized stage. One field per dish was selected and scanned sequentially every 5min for 20h at a magnification of ×10. Image analyses and measurements were performed with the ImageJ software. In the first photograph the nucleus of at least 10 cells was marked and tracked to the last photograph. Direct distance from the start point to the end point, median velocity, as well as the time the cells were moving or not were calculated.

### Invasion assay

U87 and U87^shABL^ cells (1×10^4^) were seeded in the upper compartment of 8μm-pore Boyden-like chamber (transwell, Corning) pre-coated with 3.5mg/ml Matrigel (BD Biosciences) in 200μl of RPMI supplemented with 0.5% FBS and 10μM AraC. The bottom chambers were filled with 700μl of complete RPMI media. After 24h of incubation at 37°C, non-invading cells present on the upper surface of the filter were removed with a cotton swab. The invading cells located on the underside were fixed with 4% PFA and stained with a solution of 2% Crystal Violet. Invasive ability was determined by counting cells that had migrated to the lower side of the filter. Experiments were performed in triplicate in at least 3 independent experiments.

### Cell proliferation and survival assays

U87 and U87^shABL^ cells were cultured for 2 and 5 days on glass coverslips. After pulsing with Bromodeoxyuridine (BrdU, Sigma) at a final concentration of 100μM for 6h, cells were fixed with 4% PFA, permeabilized with PBS-0.5% TritonX-100 and denatured with 2N HCl. Cells were washed with 0.1M sodium borate (pH 8.5) and incubated overnight at 4°C with primary anti-BrdU monoclonal antibody (1:300, Sigma). Cells were then incubated for 1h at room temperature with secondary donkey anti-rat Alexa 594 antibodies (1:500; Invitrogen Life Technologies). For immunostaining with AnnexinV, cells were fixed with 4% PFA, permeabilized with PBS-0.5% TritonX-100 and incubated overnight at 4°C with anti-AnnexinV (1:100; Abcys), then for 1h at room temperature with secondary goat anti-mouse Alexa 555 antibodies (1:500; Invitrogen Life Technologies). TUNEL was performed following protocol previously described [85]. Coverslips were mounted in Prolong-Gold antifade reagent DAPI.

### Soft agar assay

U87, U87^shABL^, U87^rescue^, LN19, and LN229 cells were suspended in complete RPMI media containing 0.5% agar and seeded in triplicate on 35mm dishes pre-coated with 1% agar in complete RPMI media and incubated at 37°C, 5% CO2. After 2 weeks, colonies were stained with Thiazolyl Blue Tetrazolium Bromide (MTT) (1mg/ml, Sigma), and counted. Numbers are expressed as means of triplicates.

### *In vivo* tumourigenesis assay

U87 and U87^shABL^ cells (3×10^6^) resuspended in 150μl of PBS were injected sub-cutaneously into the flank of nude mice (S/SOPF SWISS NU/NU; Charles River). Mice were then sacrificed after 4 (*n* = 11) or 8 (*n* = 4) weeks of treatment. Tumour volume was determined from caliper measurements of tumour length (L) and width (W). The formula used for tumour volume measurement was: (L x W^2^)/2. All procedures involving the use of animals were performed in accordance with the European Community Council Directive of 22 September 2010 on the protection of animals used for experimental purposes (2010/63/UE). The experimental protocols were carried out in compliance with institutional Ethical Committee guidelines for animal research (comité d'éthique pour l'expérimentation animale - Comité d'éthique de Marseille; agreement number D13-055-21 delivered by the Direction départementale des services vétérinaires - Préfecture des Bouches du Rhône).

### Tumour spheres-forming assay

Cells were cultured at a density of 2×10^4^/35mm dishes in stem cell-permissive media as previously described [58]. After 10-14 days, spheres were collected and processed for total RNA extraction. For self-renewal assays, primary spheres were dissociated after 2 weeks into single cells and re-plated at the same density as previously described [58]. Subsphere-forming assay (also called passage) was repeated every 2 weeks. After each passage, number and size of spheres were analysed.

### Statistical analysis

Results were expressed as the mean±s.e.m. Statistical significance of biological outcomes was analysed by the Mann-Whitney test when applicable, otherwise by the Student's-*t* test. Statistical significance was defined as not significant (ns): *P* > 0.05; *: *P* < 0.05; **: *P* < 0.01; ***: *P* < 0.001.

## SUPPLEMENTARY MATERIALS FIGURES AND TABLES



## Abbreviations

GBM: Glioblastoma
RTK: Receptor Tyrosine Kinase
shRNA: short hairpin RNA
RT-qPCR: real-time quantitative PCR
ZO-1: Zonal occludens-1
DMEM: Dulbecco's modified Eagle's medium
FBS: Fetal Bovine Serum
B2M: Beta-2 microglobuline
PFA: Paraformaldehyde
AraC: Cytosine arabinoside
BrdU: Bromodeoxyuridine
MTT: Thiazolyl Blue Tetrazolium Bromide
