# Supplementary material for: Coordination of signalling networks and tumorigenic properties by ABL in glioblastoma cells
Source: Oncotarget. 2016 Oct 9;7(46):74747–67. doi: 10.18632/oncotarget.12546 (PMC5342699; doi:10.18632/oncotarget.12546)
Supplement: Supplementary file 1 [file oncotarget-07-74747-s001.pdf]

Coordination of signalling networks and tumorigenic properties by ABL in glioblastoma cells

Supplementary Material

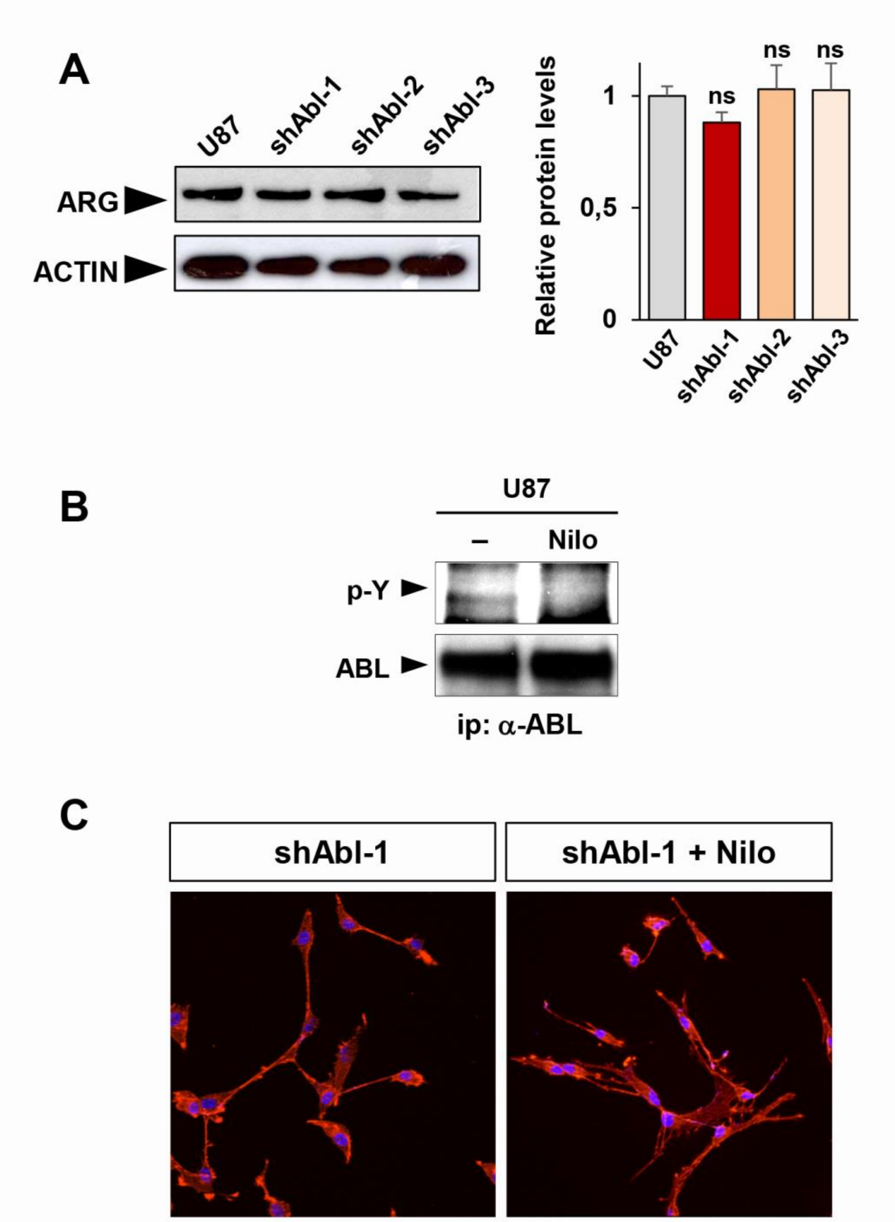

Supplementary Figure 1: Specificity of ABL targeting with shRNAs or Nilotitib treatment. (A) Representative western blot analyses (left) and quantification (right) of ARG expression levels in U87^{shABL} cells (U87 transfected with shAbl-1, shAbl-2, and shAbl-3) compared to controls (shAbl-1: 0.88±0.04 fold change; shAbl-2: 1.03±0.1 fold change; shAbl-3: 1.02±0.1 fold change). Values are expressed as means ± s.e.m. ns: not significant**. (B)** Immunoprecipitation (ip) with anti-ABL (α-ABL) antibodies followed by western-blot analysis showing constitutive phosphorylation of ABL on Tyrosine residues (p-Y) in U87 cells. After striping, blots were reprobed with anti-ABL antibodies.

Nilotinib treatment (Nilo, 3µM; 24hrs) leads to inhibition of ABL phosphorylation. **(C)** Phalloidin staining (red) of U87^{shAbl} (shAbl-1) either untreated (left) or exposed to Nilotinib (2µM; 72 hrs). Note comparable shape of cells in both conditions. Nuclei stained with DAPI are in blue.

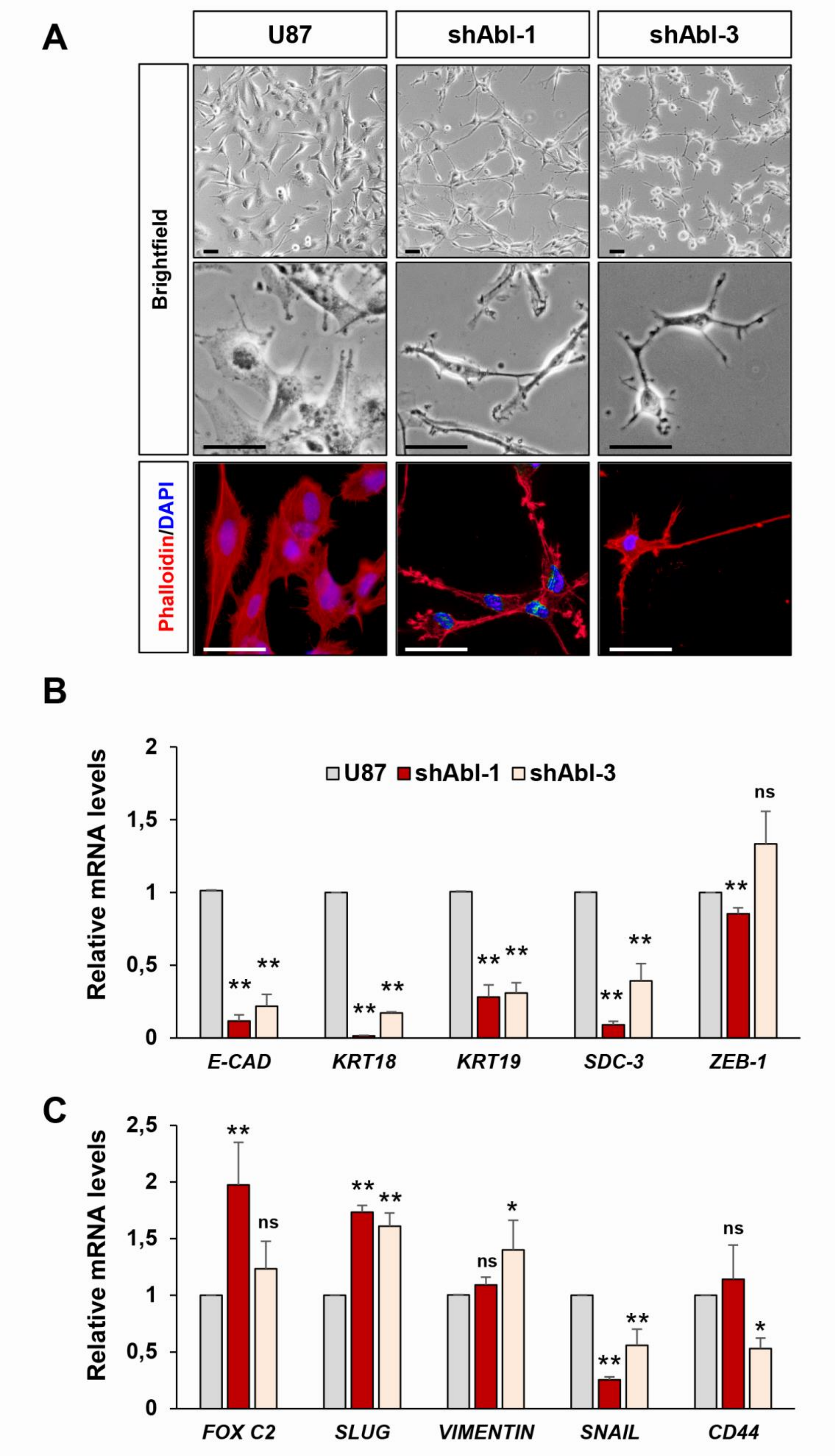

Supplementary Figure 2: Analysis of morphology and epithelial-mesenchymal markers in U87 cells with ABL impairment. (A) Brightfield (top and middle) and phalloidin staining (red; bottom) images

of U87 and U87^{shAbl} (shAbl-1 and shAbl-3) cells. Note that ABL inhibition using shAbl-3 leads to drastic morphological changes compared to control cells. Nuclei stained with DAPI are in blue. Scale bars: 50µm. **(B, C)** RT-qPCR analysis of *E-cadherin (E-CAD), Cytokeratin-18* (*KRT18*), *Cytokeratin-19* (*KRT19*), *Syndecan-3 (SDC-3), ZEB-1, FOX C2, SLUG, VIMENTIN, SNAIL,* and *CD44* in U87 and U87^{shAbl} (shAbl-1 and shAbl-3) cells. Note that for appropriate comparisons with shAbl-3 results, qualitative and quantitative data corresponding to U87 and shAbl-1 cells in all Supplementary Figures are the same as those reported in Figures. Results are the mean of three independent experiments. Values are expressed as means ± s.e.m. ns: not significant; * *P*<0.05; ** P<0.01.

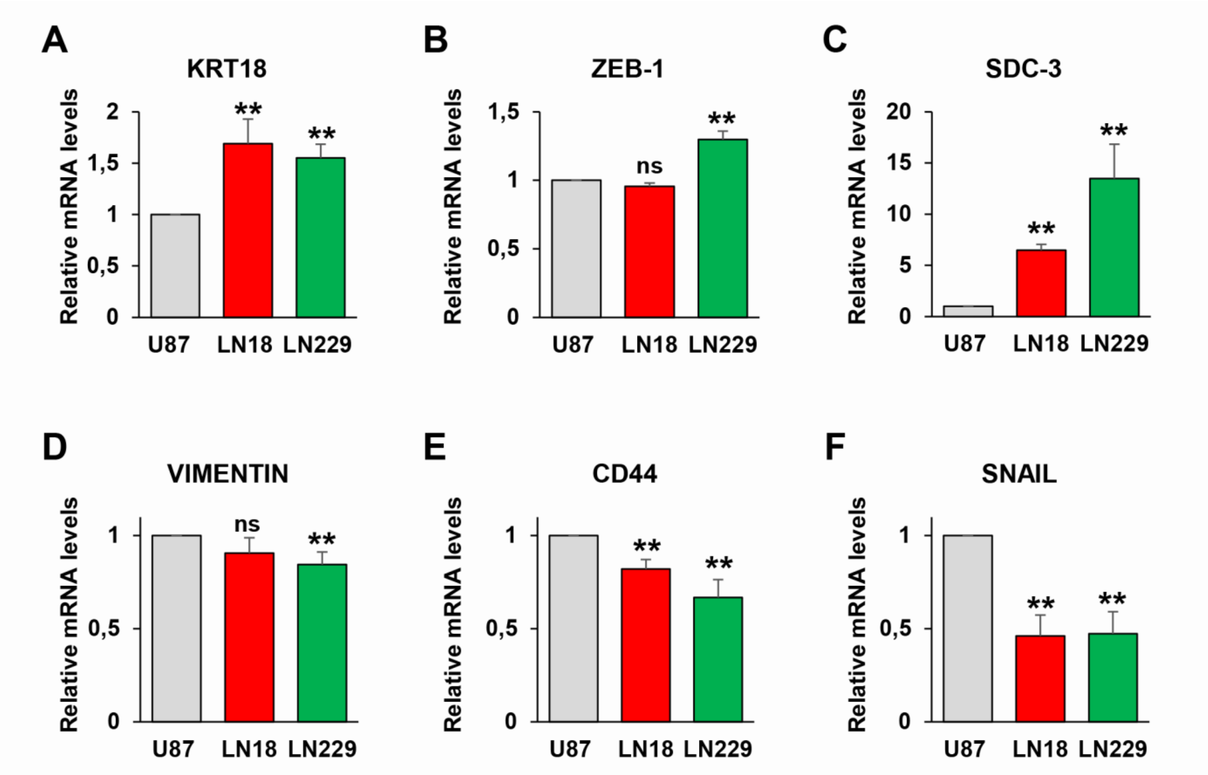

Supplementary Figure 3: (A-F) RT-qPCR analysis showing expression levels of epithelial (A-C) and mesenchymal (D-F) markers in U87, LN18, and LN229 cells. Each experiment was done at least in triplicates. Values are expressed as means ± s.e.m. ns: not significant; ** *P* 0.01.

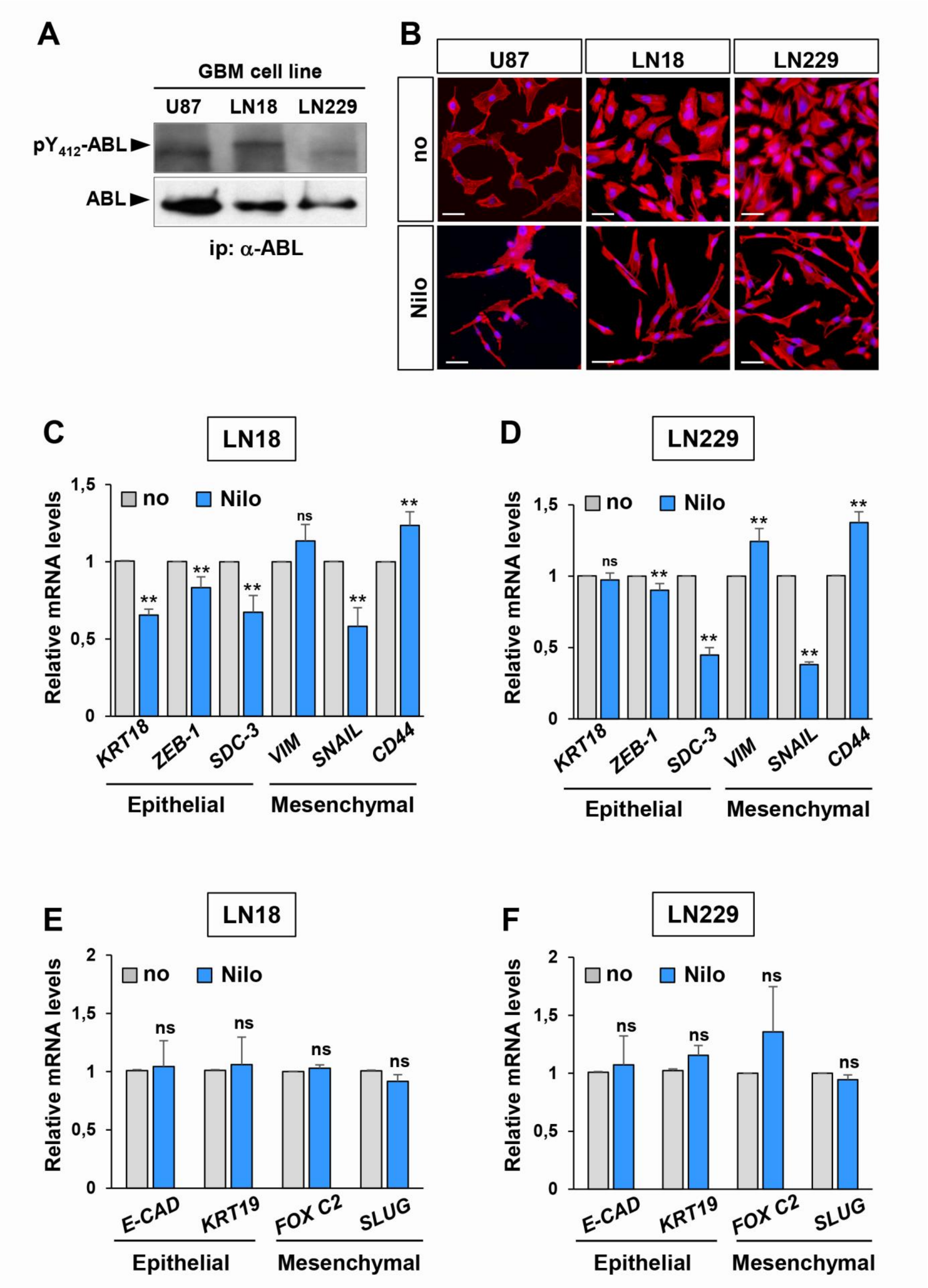

Supplementary Figure 4: Morphological and molecular analyses in LN18 and LN229 cells with ABL impairment. (A) Protein lysates of U87, LN18, and LN229 cell lines were subjected to immunoprecipitation (ip) with anti-ABL (ip: α-ABL) antibodies followed by western blot analysis with

phospho-ABL (pY_{412}-ABL). After striping, blots were reprobed with anti-ABL antibodies. **(B)** Phalloidin staining (red) images of untreated U87, LN18, and LN229 cells exposed to Nilotinib (2µM; 72 hrs). Note that ABL inhibition leads to the acquisition of a fusiform cell shape. Nuclei stained with DAPI are in blue. Scale bars: 50µm. **(C - F)** RT-qPCR analysis showing changes in the expression levels of epithelial and mesenchymal markers in LN18 (C and E) and LN229 (D and F) cells exposed to Nilotinib (5µM) compared to control cells. CD44 is considered as a mesenchymal and stemness marker, according to the cellular context and the culture conditions. In this experimental setting, CD44 levels evidence mesenchymal rather than stemness traits. Note that some epithelial and mesenchymal markers are changed in Nilotinib-treated cells (C and D), other are not affected (E and F). Each experiment was done at least in duplicate. Values are expressed as means ± s.e.m. ns: not significant; ** *P* 0.01.

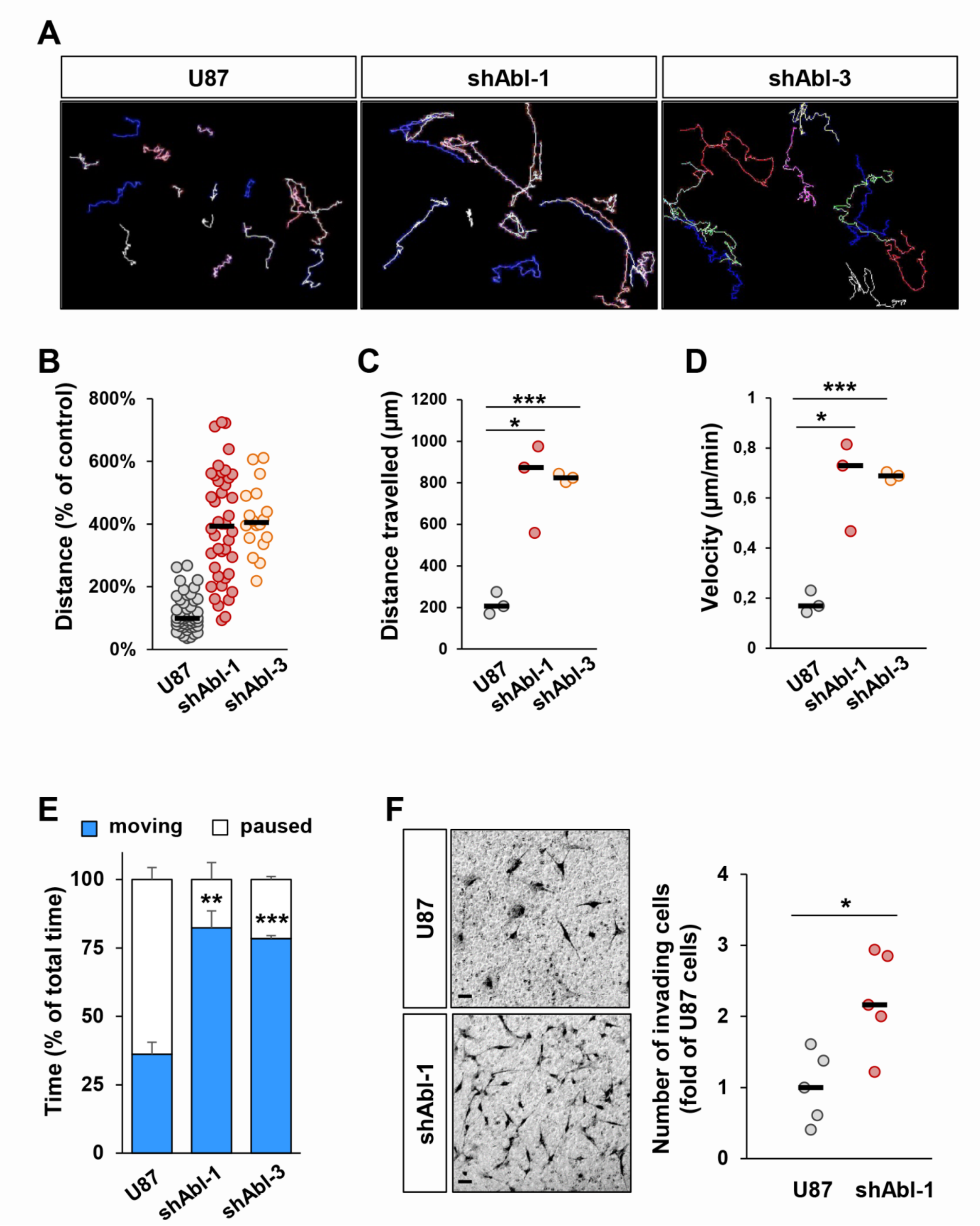

Supplementary Figure 5: Increased migration and invasion properties of U87 cells with impaired Abl. (A) Representative images of migration paths of U87, shAbl-1, and shAbl-3 cells analysed by time-lapse videomicroscopy (n=3). **(B-E)**. Quantification of time-lapse videomicroscopy showing the distance the cells travelled expressed as percentage of the distance travelled by control cells (B, each dot represents a single cell analysed), the total distance the cells travelled (C, each dot represents the mean of independent experiments; shAbl-3: 824.5±11.2µm), the mean velocity of the cells during the

20hrs of recording (D, each dot represents the mean of independent experiments; shAbl-3: 0.68±0.01µm/min), and the time percentage the cells spent moving versus paused (E). **In E** values are expressed as means ± s.e.m. * *P* 0.05; ** *P* 0.01; *** *P*<0.001. **(F)** Invasion assays were performed with Transwell chamber pre-coated with matrigel (left) and quantified by counting cells that migrated through the membrane (right; n=5; shAbl-1: 2.16±0.31 fold versus controls). Each dot represents the mean of independent experiments done in triplicate. Data show the higher invasive capacity of $U87^{shAbl}$ cells compared to controls. Values are expressed as means ± s.e.m. * *P*<0.05.

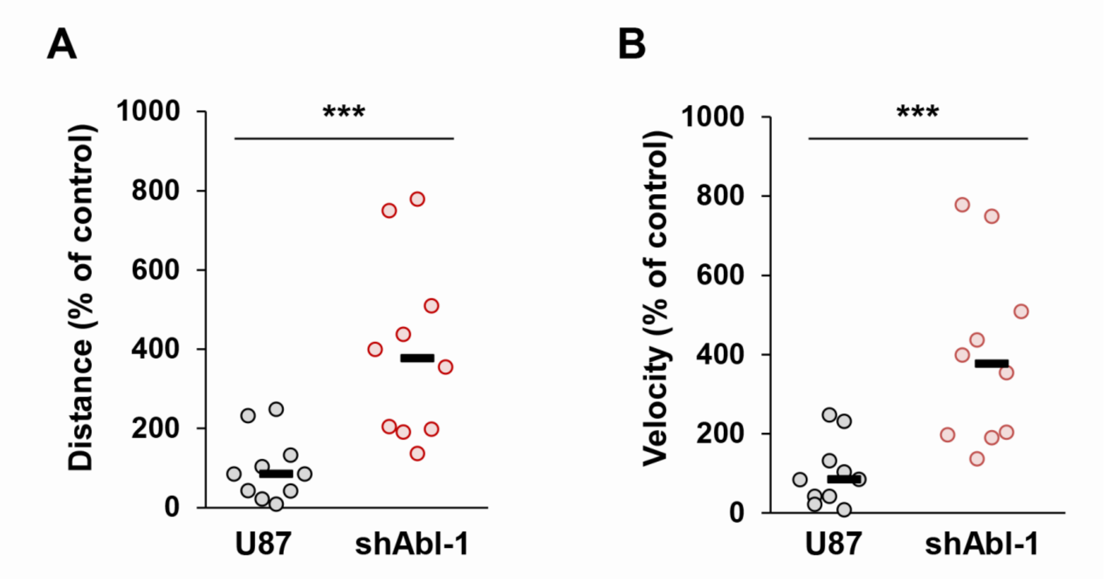

Supplementary Figure 6: Analysis of migration capacities of $U87^{shABL}$ cells in the absence of AraC. (A and B) Quantification of time-lapse videomicroscopy in the absence of AraC showing the total distance the cells travelled (A) and the mean velocity of cells (B) during the 20hrs of recording. Values are expressed as means ± s.e.m. *** P <0.001.

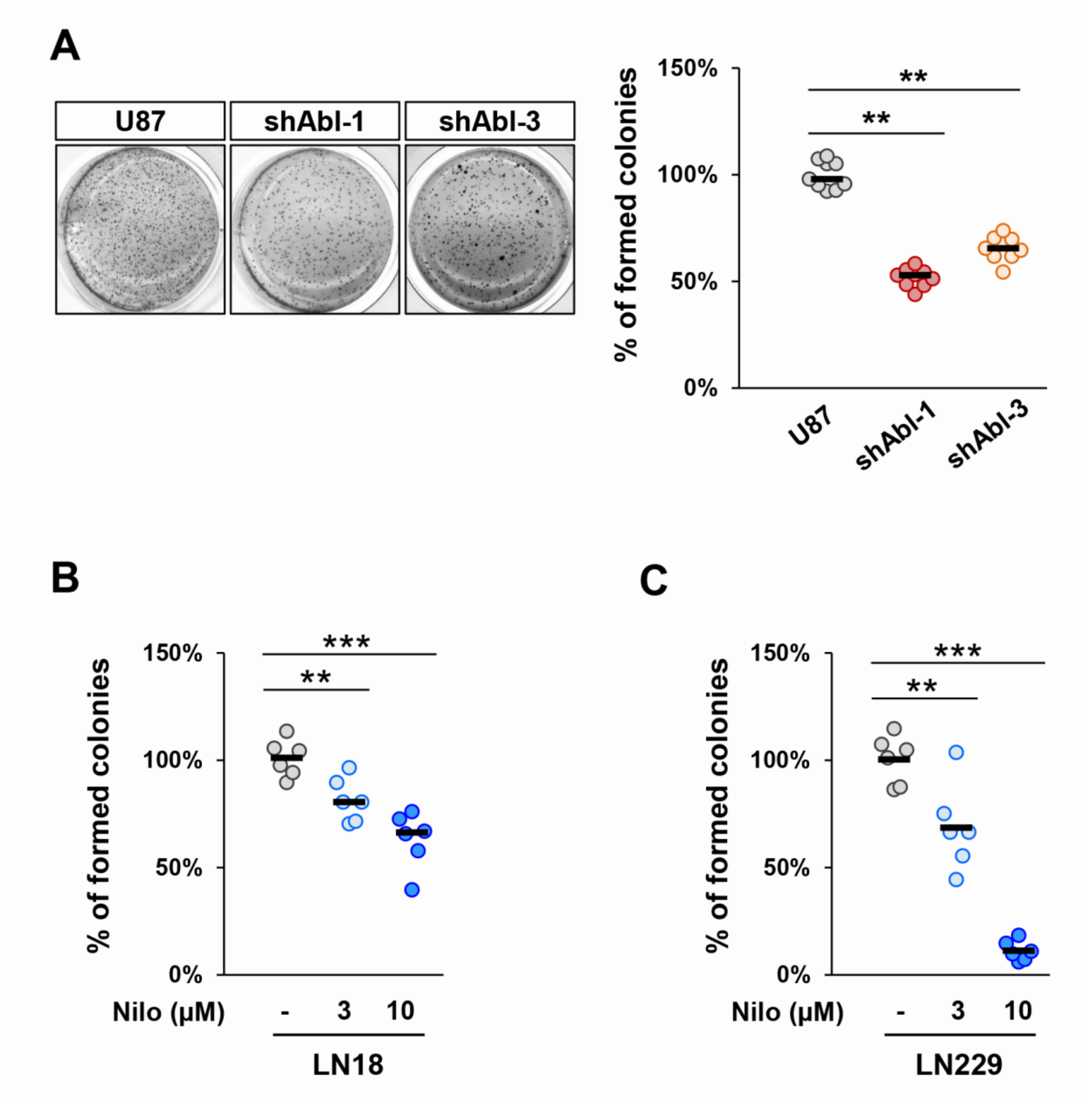

Supplementary Figure 7: Tumorigenic properties of GBM cells are impaired by ABL inactivation. (A) Anchorage-independent growth assay showing reduced in vitro tumorigenic properties of shAbl-3 cells (65.5±1.9%). Representative images (left) and quantification (right) are shown. **(B)** Anchorage-independent growth assays showing reduced in vitro tumorigenic properties of LN18 and LN229 cell lines exposed to Nilotinib treatment (Nilo, 3µM and 10µM). Each dot represents the mean of independent experiments. Values are expressed as means ± s.e.m. ** *P*<0.01, *** *P*<0.001.

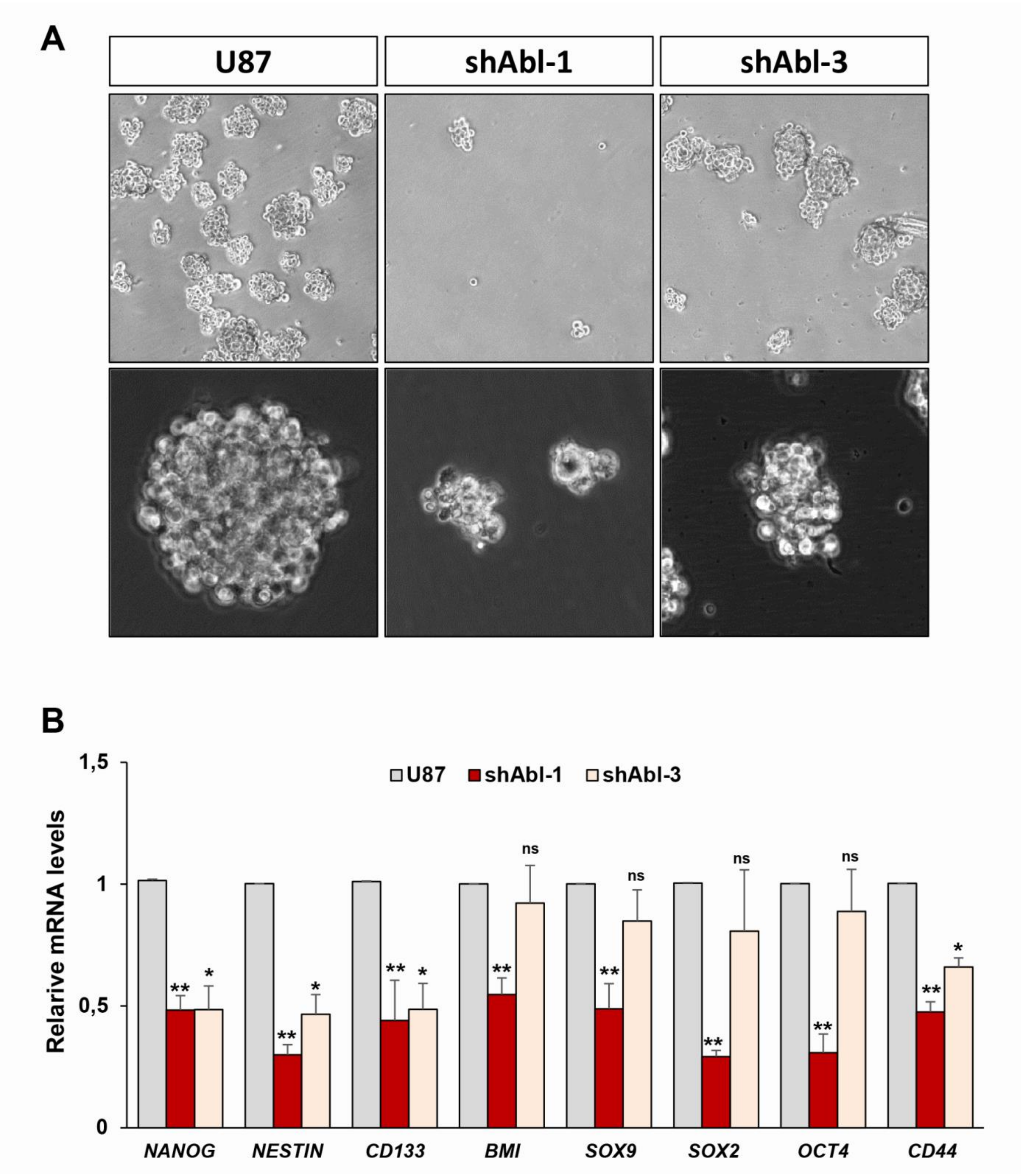

Supplementary Figure 8: Permanent Abl ablation interferes with neurosphere formation and expression of self-renewal markers in GBM cells. (A) Representative images of spheres derived from U87, shAbl-1, and shAbl-3 cells. **(B)** RT-qPCR analyses showing the repression of pluripotent markers (*NANOG, NESTIN, CD133, BMI, SOX9, SOX2, OCT4*, and *CD44*) in shAbl-3 compared to U87 spheres. Data correspond to biological triplicates. Values are expressed as means ± s.e.m. ns: not significant; * *P* 0.05; ** *P* 0.01.

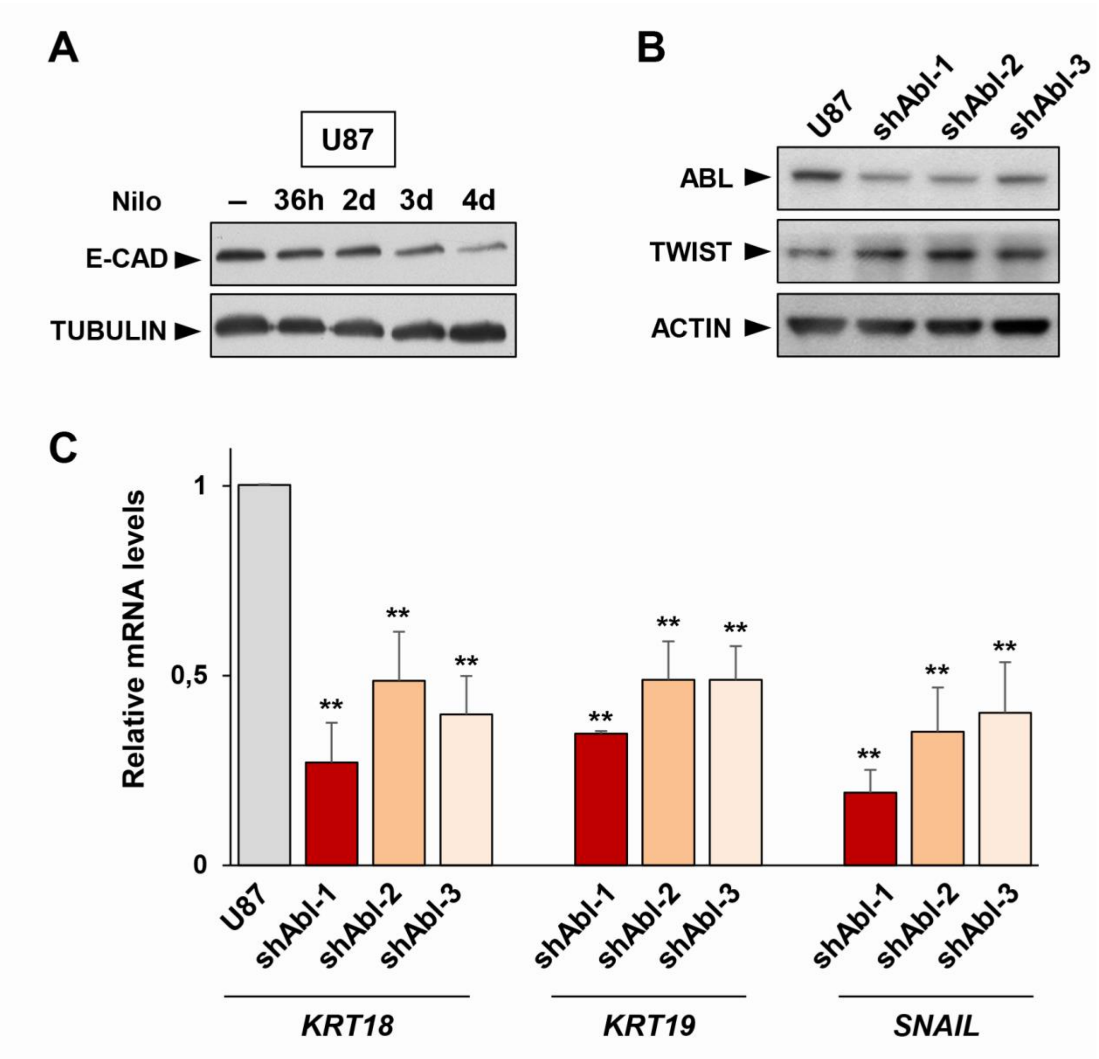

Supplementary Figure 9: (A) Time-course analysis by western blots of E-Cadherin (E-CAD) expression after Nilotinib treatment (5µM). TUBULIN was used as loading control. **(B)** Western blots of protein extracts from cells transfected with shRNA targeting plasmids showing, already after 48 hrs, down-regulation of ABL and up-regulation of TWIST-1 (TWIST). **(C)** RT-qPCR analysis of mRNA levels from cells used in panel B, showing *Cytokeratin-18* (*KRT18*), *Cytokeratin-19* (*KRT19*), and *SNAIL* expression levels. Values are expressed as means ± s.e.m. ** *P* 0.01.

Supplementary Table 1: Sequence of the three shRNAs targeting ABL is shown, with their identities versus human ABL and human ARG. In the right column, the number of nucleotides over the total that are identical to human ARG is reported as well as the overall percentage of identity.

Abl shRNA	sequence	Identities vs human ABL	Identities vs human ARG
shAbl-1	5'-AGGTGAAAAGCTCCGGGTC-3'	100%	12/19 (63%)
shAbl-2	5'-ATGCTTAGAGTGTTATCTC-3'	100%	15/19 (79%)
shAbl-3	5'-AATGGAGCGTGGTGATGAG-3'	100%	9/19 (47%)

Supplementary Table S2: Primer sequences used for RT-qPCR analyses.

Target Gene	**Forward primer (5'-3')**	**Reverse primer (5'-3')**
ABL1 (ABL)	CCAGGTGTATGAGCTGCTAGAG	GTCAGAGGGATTCCACTGCCAA
B2M	CCACTGAAAAAGATGAGTATGCCT	CCAATCCAAATGCGGCATCTTCA
BMI1 (BMI)	GGTACTTCATTGATGCCACAACC	CTGGTCTTGTGAACTTGGACATC
CD133	CACTACCAAGGACAAGGCGTTC	CAACGCCTCTTTGGTCTCCTTG
CD44	CCAGAAGGAACAGTGGTTTGGC	ACTGTCCTCTGGGCTTGGTGTT
Cytokeratin 18 (KRT18)	GCTGGAAGATGGCGAGGACTTT	TGGTCTCAGACACCACTTTGCC
Cytokeratin 19 (KRT19)	AGCTAGAGGTGAAGATCCGCGA	GCAGGACAATCCTGGAGTTCTC
E-Cadherin (E-CAD)	GCCTCCTGAAAAGAGAGTGGAAG	TGGCAGTGTCTCTCCAAATCCG
FOX C2	TCACCTTGAACGGCATCTACCAG	TGACGAAGCACTCGTTGAGCGA
NANOG	CTCCAACATCCTGAACCTCAGC	CGTCACACCATTGCTATTCTTCG
NESTIN	TCAAGATGTCCCTCAGCCTGGA	AAGCTGAGGGAAGTCTTGGAGC
OCT4	CCTGAAGCAGAAGAGGATCACC	AAAGCGGCAGATGGTCGTTTGG
SNAIL1 (SNAIL)	*CGAAAGGCCTTCAACTGCAAAT*	*ACTGGTACTTCTTGACATCTG*
SNAIL2 (SLUG)	ATCTGCGGCAAGGCGTTTTCCA	GAGCCCTCAGATTTGACCTGTC
SOX2	GCTACAGCATGATGCAGGACCA	TCTGCGAGCTGGTCATGGAGTT
SOX9	AGGAAGCTCGCGGACCAGTAC	GGTGGTCCTTCTTGTGCTGCAC
Syndecan-3 (SDC-3)	CTCCTGGACAATGCCATCGACT	TGAGCAGTGTGACCAAGAAGGC
TWIST-1 (TWIST)	GCCAGGTACATCGACTTCCTCT	TCCATCCTCCAGACCGAGAAGG
VIMENTIN	AGGCAAAGCAGGAGTCCACTGA	ATCTGGCGTTCCAGGGACTCAT
ZEB-1	GGCATACACCTACTCAACTACGG	TGGGCGGTGTAGAATCAGAGTC
